# Concordance between subjective and objective measures of infant sleep varies by age and maternal mood: Implications for studies of sleep and cognitive development

**DOI:** 10.1016/j.infbeh.2021.101663

**Published:** 2022-02

**Authors:** L.K. Gossé, F. Wiesemann, C.E. Elwell, E.J.H. Jones

**Affiliations:** a Centre for Brain and Cognitive Development, Birkbeck, University of London, London, United Kingdom; b Research & Development, Procter & Gamble, Schwalbach am Taunus, Germany; c Department of Medical Physics and Biomedical Engineering, Biomedical Optics Research Laboratory, University College London, London, United Kingdom

**Keywords:** Infant sleep, Cross-method concordance, Actigraphy, Parent-report, General development, Maternal stress

## Abstract

Infant habitual sleep has been proposed as an important moderator of development in domains such as attention, memory or temperament. To test such hypotheses, we need to know how to accurately and consistently assess habitual sleep in infancy. Common assessment methods include easy to deploy but subjective parent-report measures (diary/sleep questionnaire); or more labour-intensive but objective motor movement measures (actigraphy). Understanding the degree to which these methods provide converging insights is important, but cross-method agreement has yet to be investigated longitudinally. Moreover, it is unclear whether concordance systematically varies with infant or maternal characteristics that could represent confounders in observational studies. This longitudinal study (up to 4 study visits/participant) investigated cross-method concordance on one objective (7-day actigraphy) and three commonly used subjective (7-day sleep diary, Brief Infant Sleep Questionnaire, Sleep & Settle Questionnaire) sleep measures in 76 typically developing infants (age: 4–14 months) and assessed the impact of maternal characteristics (stress, age, education) and infant characteristics (age) on cross-method concordance. In addition, associations between objective and subjective sleep measures and a measure of general developmental status (Ages & Stages Questionnaire) were investigated. A range of equivalence analyses (tests of equivalence, correlational analyses, Bland-Altman plots) showed mixed agreement between sleep measures. Most importantly, cross-method agreement was associated with maternal stress levels and infant age. Specifically, agreement between different measures of night waking was better for mothers experiencing higher stress levels and was higher for younger than older infants; the reverse pattern was true for day sleep duration. Interestingly, objective and subjective measures did not yield the same patterns of association with developmental domains, indicating that sleep method choice can influence which associations are found between sleep and cognitive development. However, results converged across day sleep and problem-solving skills, highlighting the importance of studying day sleep in future studies. We discuss implications of sleep method choice for investigating sleep in the context of studying infant development and behaviour.

## Highlights

• A range of equivalence analyses showed mixed agreement between subjective and objective sleep measures.
• Cross-method agreement was associated with maternal stress levels and infant age.
• Objective and subjective measures did not yield the same patterns of association with developmental domains except for day sleep duration.

## 1 Introduction

Newborn infants can sleep up to 20 h a day, with sleep only gradually declining over the course of infancy, childhood and adolescence (Bathory and Tomopoulos, 2017, Gorgoni et al., 2019, Iglowstein et al., 2003, Lewandowski et al., 2011). However, there are substantial individual differences in infant sleep consolidation; for example, night sleep duration in 6-month-old infants varies from 10 to 18 h a night between different babies (Iglowstein et al., 2003). Similarly, some infants wake up to 5 times a night at this age, while others wake only once (Iglowstein et al., 2003). It has been proposed that individual differences in infant sleep could influence neurocognitive development (e.g., Friedrich, Wilhelm, Mölle, Born, & Friederici, 2017; Friedrich, Mölle, Friederici, & Born, 2018; Mason, Lokhandwala, Riggins, & Spencer, 2021; Pisch, Wiesemann, & Karmiloff‐Smith, 2019). To understand the role of sleep in early development, we need robust and reliable measures of infant sleep that are not confounded by characteristics of the child and family.

### 1.1 Sleep and development

Emerging evidence supports an association between individual differences in infant sleep and aspects of general cognitive development (e.g., Gibson, Elder, & Gander, 2012; Horváth & Plunkett, 2016; Pisch et al., 2019; Sun et al., 2016; Mason et al., 2021 for review). In newborns, longer longest sleep period measured with a pressure-sensitive mattress correlated with better scores on the Bayley Scales, a tool to assess developmental status, at 6 months (Freudigman & Thoman, 1993). In older infants, better infant sleep (i.e., better sleep efficiency and/or longer sleep duration measured with actigraphy) has been shown to be positively related to later mental development at 10 months (Scher, 2005), subscales of the developmental Age & Stages Questionnaire at 11–13 months (Gibson et al., 2012) and also better problem-solving skills in a motor problem-solving task (Horger, DeMasi, Allia, Scher, & Berger, 2021a). Other studies have examined the influence of sleep on subdomains of cognition, showing positive effects on memory (e.g., Friedrich et al., 2017, Friedrich et al., 2018; Friedrich, Mölle, Friederici, & Born, 2020) or attention (Geva, Yaron, & Kuint, 2016). Longitudinal studies demonstrated that early sleep characteristics like sleep transitions measured with a pressure sensitive mattress relate to parent-reported infant development in the first year (Judge, Chang, & Lammi-Keefe, 2015). It has also been shown that worse parent-reported (using the Brief Infant Sleep Questionnaire) sleep quality (i.e., longer night awakings) is related to slower later cognitive development as measured by the Bayley Scales, especially during late infancy and early toddlerhood (Sun et al., 2018). In conclusion, a range of studies have indicated that individual differences in sleep may relate to individual differences in early cognitive development (for review: Mason et al., 2021).

These positive associations should be contextualised with several failures to observe similar effects with the same measures (actigraphy and Bayley scales) and lack of support from studies using different measures (e.g., parent-report measures). Neither Spruyt et al. (2008) - using actiwatches and the Bayley scales - nor Mindell and Lee (2015) - using the parent-report measures Ages & Stages Questionnaire and the Brief Infant Sleep Questionnaire - observed the association between infant sleep and cognition reported by Scher and colleagues (2005) (Mindell and Lee, 2015, Scher, 2005, Spruyt et al., 2008). Though disparities might be explained by differing infant ages across studies as the longitudinal investigations by Mindell and Lee (2015) and Spruyt et al. (2008) did not study the 10-month-old age group that Scher and colleagues (2005) focused on. Additionally, Camerota, Gueron-Sela, Grimes, and Propper (2020) did not find a direct association between cognition, as measured by the Bayley-III, an infant attention task and any measures of sleep, as assessed by actigraphy and videosomnography in 6-month-old infants. Further, the role of night vs day sleep seems to be scarcely studied, though some evidence suggests more daytime naps in 7-month-old infants predicts increased vocabulary growth in early childhood (Horváth & Plunkett, 2016). Lastly, an intriguing recent study found that sleep with no interruptions (no sleep fragmentation) in newly walking infants was associated with *worse* performance on a motor problem-solving task, highlighting the importance of studying sleep and development across different ages longitudinally (Horger et al., 2021a).

This disagreement between findings might be explained by several key limitations in the literature that limit our ability to integrate these studies to form a coherent picture of the role of sleep in early development. These include different studies investigating slightly different age groups; using different scales to assess aspects of cognition, e.g., Bayley Scales vs. Ages & Stages Questionnaire; and critically, substantial variation in how sleep is measured, e.g., actigraphy vs. questionnaires.

### 1.2 Sleep assessment measures

As described above, infants exhibit large intra- and interindividual variability in sleep patterns (Lewandowski et al., 2011, Gorgoni et al., 2019). Moreover, periods of sleep also occur during both the day and night, and many young infants often sleep during the day in places other than their beds (e.g., prams or slings). Thus, accurate measures of sleep require lengthy periods of data acquisition across multiple settings and times of day. Using the adult gold-standard of spending one or two nights in a polysomnography (PSG) lab is therefore unlikely to capture the natural pattern of sleep in the infant, although it does allow investigators to probe the neural activity that occurs during sleep in babies and classify sleep stages reliably (Guilleminault, 2005). Thus, to measure infant sleep holistically researchers are turning towards field-based approaches.

The most common field-based approach used in infant research is parent-report (e.g., Mindell et al., 2016; Sadeh, Mindell, Luedtke, & Wiegand, 2009; Sadeh, Alster, Urbach, & Lavie, 1989). Parents are important sources of information about early child development as they observe their child across multiple contexts, and can take into account important contextual information when judging sleep patterns. Commonly used parent-report sleep questionnaires include the Brief Infant Sleep Questionnaire (BISQ) (Sadeh et al., 1989, Sadeh, 1996) that has shown good validity and reliability in previous studies (Sadeh, 2004, Mindell et al., 2019 further information see below Section 2.2.2.). Alternative approaches are sleep diaries/logs or app-based reporting methods (e.g., Mindell et al., 2016). Parent-report methods are easy to use; most questionnaires are brief and scalable and can also be easily integrated over a longer period of time, e.g., in longitudinal studies. Moreover, they are less invasive than device-based methods (like actigraphy or PSG) and may potentially yield better representation through reduced data loss. However, parent opinion of their child’s sleep may be influenced by parental characteristics (de Los Reyes & Kazdin, 2005). Additionally, questionnaires often fail to capture the variability in sleep patterns apparent in sleep patterns of young infants.

An alternative approach is to use objective recording devices in the home. In recent decades, actigraphy has become more popular in infant research as an option to collect ecologically valid and non-biased data. The small, watch-like device may be worn at home for several consecutive days, weeks and even decades (Borbély, Rusterholz, & Achermann, 2017), assessing real-life sleep-wake patterns and circadian rhythms that are not distorted by laboratory settings. Actigraphy refers to a continuous measurement of human activity and rest periods using a tri-axial accelerometer. As an objective measure, actigraphy may be able to circumvent some of the inherent biases of parent-report (although notably many analytic approaches include adjustment of actigraphy data by diary-based reports of sleep and wake times from parents). With rapidly developing wearables technology in the private sector (e.g., Fitbit), precise, wearable sleep assessments may soon be widely available. Moreover, the small, unobtrusive, watch-like device is a good tool for sleep studies in infant populations and clinical populations such as children with autism spectrum disorder (Yavuz-Kodat et al., 2019). Lastly, it is likely less influenced by other maternal or infant characteristics (like mood or temperament) than parent-report measures (Sadeh, 2011). However, although widely used, actigraphy should not be automatically considered to be ‘better’ than parent-report. Actigraphy has been shown to occasionally under- or overestimate true sleep (Tsai et al., 2009, Sadeh, 2011, Galland et al., 2018), particularly in situations where infants sleep in non-bed settings (e.g., pram, sling, Meltzer, Montgomery-Downs, Insana, & Walsh, 2012). As accelerometers, actigraphs do not discriminate well between external movements (such as a jostling pram) and real movements of the subject (Tsai et al., 2009). In these cases, the patterns of motion can cause algorithms to incorrectly identify periods of sleeping as waking, and vice versa. Further, actigraphy requires infants to be comfortable with wearing a device over long periods, something that may contribute to differential drop-out based on infant sensitivity. Research-grade devices are not cheap, and thus the scalability of their use is more limited than parent-report alone. This is important when considering individual differences, where large samples are necessary to detect robust effects. In summary, both parent-report and actigraphy can provide important information about infant sleep, but both have limitations that must be considered when interpreting results.

### 1.3 Comparability between objective and subjective measures

Understanding the concordance between objective and subjective measures is important because it reveals the degree of generalisation across methods. Both parent-report and actigraphy-based measures are likely to have systematic sources of error, and consequently areas of cross-method consistency are especially informative. Previous studies that have directly compared actigraphy and questionnaires have shown that parents may over- or underestimate sleep duration relative to actigraphy-based measures (Camerota et al., 2018a, de Los Reyes and Kazdin, 2005). Consistency of parent estimates of infant sleep with actigraphy-based measures may be influenced by a range of variables that include parents’ personal characteristics, habits and schedule, sleep location (co-sleeping vs. own room), infant feeding method (breastfeeding vs. bottle, Rudzik, Robinson-Smith, & Ball, 2018) and stress level or fatigue causing difficulty in recording and remembering infants sleep-wake times. This is based on prior research into consistency of parent-report measures of development and/or sleep (Camerota et al., 2018a, de Los Reyes and Kazdin, 2005).

A few recent studies have compared different methods of sleep assessment in infants. Most notably, a study by Camerota et al., 2018a used one subjective method and two objective methods (videosomnography and actigraphy) in 90 3-month-old infants. Both sleep quality and quantity were measured. Correlations for sleep methods were good for sleep schedule and moderate for night wakening. Actigraphy and sleep diaries coincided better than either of those measures coincided with videosomnography. Another study by Tikotzky and Volkovich (2019) only assessed night waking number and duration using two subjective (BISQ, sleep diaries) and one objective (actigraphy) method. The researchers report greater actigraphy-assessed wake after sleep onset (WASO) at 6, 12, and 18 months of age but no differences at 3 months of age; overall there was a higher number of night wakings reported by subjective measures. Agreement between methods was higher for the younger age group than the older age groups, and while correlations were high between the measures, agreement between methods (as measured by Krippendorfs alpha) was poor, especially for BISQ and actigraphy. One exception was that Bland-Altman (BA) plots showed that agreement for WASO was good between diary and actigraphy for 12- and 18-months-olds. These findings contrast with earlier findings showing that actigraphy overestimated night wakings compared to diary, and night sleep duration was different between the methods across a sample 5.5- to 8-month-old infants (Hall, Liva, Monynihan, & Saunders, 2015). However, they are in line with findings by Müller et al. (2011) that found good agreement between sleep diary and actigraphy in 1- to 9-month-old infants (Müller, Hemmi, Wilhelm, Barr, & Schneider, 2011). A key difference between the studies is that Müller and colleagues (2011) pooled the large age range and did not differentiate between age groups. Depending on the age group distributions this could either increase or decrease cross-method agreement. More recently Horger, Marsiliani, DeMasi, Allia, and Berger (2021b) published a comparison of three different sleep measures (videosomnography, actigraphy and parent-report) in a small, longitudinal sample (N = 9) where sleep parameters showed different developmental trajectories depending on which sleep technique was used. For example, parent-report showed quadratic associations between wake episodes and age whereas videosomnography showed a linear association.

In conclusion, while some studies show consistencies across actigraphy and parent-report measures other studies show differences across measures. The varied cross-method concordance observed across studies likely reflects multiple factors (including infant age, questionnaire measures selected and equipment used; Schoch, Kurth, & Werner, 2021). However, there may also be systematic differences in measure concordance related to aspects of the parents and infants studied. These considerations are crucial to consider in studies that make comparisons in infant sleep between different ages, or between groups defined by different infant or parent risk factors (like anxiety or depression). This aspect is often not taken into account or studied when cross-method concordance is investigated.

### 1.4 Current study

Overall, previous work has yielded mixed findings on the consistency between different ways of assessing infant sleep. Some studies show good agreement and other studies show poor agreement between subjective (diary/questionnaire) and objective (actigraphy) measures. Studies that compare agreement of sleep measures across the entire first year of life are lacking. Tracking different methods side-by-side longitudinally in typically developing infants is crucial to identify whether objective or subjective methods might provide different consistency across time points. Moreover, few studies have examined the factors that might influence agreement between different sleep measures (e.g., Tikotzky & Volkovich, 2019), such as infant age or parental mood and this constitutes a key added value in this study compared to prior studies published to date on this topic. Lastly, studies so far have not investigated how method choice could impact observed associations between infant sleep and other aspects of cognitive development.

By investigating general development in relation to both objectively and subjectively measured sleep in a large accelerated longitudinal design, we aim to characterise how the concordance between different measures of sleep (actigraphy and parent-report sleep measures) varies with infant age and with parental characteristics (such as maternal stress); and whether there are consistent and stable associations with other measures of infant cognitive development (as assessed by the parent-report Ages & Stages Questionnaire). As a similar study (Pisch, 2015) with comparable sample found an association between sleep parameters and Ages & Stages general development, we expect to find similar associations here. This will provide critical input into our interpretation of the current literature on infant sleep by also comparing different subjective measures in detail in a large sample size and by extending this research to take a look what might drive the disparities in the literature. Our longitudinal study focused on the first year of life as this is a time period where many changes happen in sleep as well as development.

We report four main analyses in the current paper in addition to descriptive statistics. Analysis 1 examined the concordance between subjective and objective sleep measures through a) correlational analyses (incl. ICC analysis); b) Bland-Altman plots; and c) equivalence testing. Analysis 2 investigated how maternal and infant characteristics associate with concordance between different sleep measures. Analysis 3 investigated the consistency of age-related changes in sleep parameters across the different methodologies. Lastly, Analysis 4 investigated cross-method concordance in association with a developmental measure.

## 2 Material and methods

### 2.1 Procedure

The study used an accelerated longitudinal design, with participants enroled at 4, 6, 8, 10, 12, and 14 months of age. Accelerated longitudinal design refers to a longitudinal design that starts several waves of recruitment at multiple ages (Fields, 2005). This study was approved by the Department of Psychological Sciences, Birkbeck, University of London, Ethics committee in accordance with the Declaration of Helsinki and conducted at the German Innovation Center, Procter & Gamble, Schwalbach am Taunus, Germany. All parents provided informed consent for the participation of their infant. Parents initially attended the lab for an in-person visit at which some experimental measures were completed (not reported here). After administration of consent, parents were shown by the researcher how to attach and take off the actigraph to the infant’s ankle to ensure accurate measurement. Parents then took home the key sleep questionnaires and questionnaires assessing their stress level and socio-economic status, a sleep diary and the actigraph (for details see below). Sleep diary and actigraphy data were collected for seven consecutive days simultaneously. After 7 days sleep diary, actigraphy and questionnaire package was returned to the researcher.

#### 2.1.1 Participants

The present sample included 76 typically developing, term-born (GA > 37 weeks) infants (42 female) without a (familial) medical history of sleep, neurodevelopmental or neuropsychiatric disorders assessed by parent-report. The age range at recruitment ranged from 4 to 14 months (mean age: 282 days, SD = 92 days, range in days: 116–456 days). Participants were tested every 2 months for 6 months or until they were 14 months old. Exclusion criteria were a family history of neurodevelopmental and/or sleep disorders, premature birth (< 37 GA), and plans to move away from the Frankfurt area in the near future.

### 2.2 Sleep measures

#### 2.2.1 ActiGraph wGT3X-BT

We used an ActiGraph wGT3X-BT from ActiGraph Corp. Based on the previous literature (Meltzer et al., 2012; Pisch, 2015; Sadeh, Sharkey, & Carskadon, 1994)), sampling frequency was set at 60 Hz, the device to zero-crossing mode, and at least five 24-h segments of data were required for the data set to qualify for inclusion in the final actigraphy dataset. The actigraphs were initialised for the whole duration of the study at the lab visit without caregivers having to turn it on or off. Caregivers were encouraged to let the infant wear the actigraph for 24 h for 7 days and asked to record duration and dates for when the actigraph was removed (used in data analysis). Strategies to improve compliance included instruction sheets for use of the actigraph and personal communication with parents during the study week (see SM for instruction sheet given to parents regarding the sleep data).

#### 2.2.2 Brief Infant Sleep Questionnaire (BISQ)

The BISQ is a short, standardised screening questionnaire that assesses habitual infant sleep patterns of the week prior to completion date by asking about classical sleep parameters such as sleep duration or number of night waking but also about parental perception of sleep and sleep routines. BISQ completion time is 5–10 min. Good validity was established by Sadeh (2004) originally compared to actigraphy and sleep diaries and more recently by Mindell et al. (2019). Based on Pisch (2015), one question on sleep rituals and six questions on parental sleep and parental perception of infant sleep were included. Parents were asked to fill out the BISQ at the end of the week of actigraphy and sleep diary thinking about their infant’s sleep in the week prior.

#### 2.2.3 Sleep and Settle Questionnaire (SSQ)

Instructions to parents regarding the SSQ were the same as for the BISQ. The SSQ is a 34-item questionnaire that assesses infant sleep and settling behaviour, as well as level of parental concern over these behaviours of the week prior to completion date (Matthey, 2001). It is thus a screening tool for identifying infants with difficulties of falling asleep and staying asleep as well as their daytime behaviour (Lewandowski et al., 2011, Matthey, 2001). Test–rest reliability ranged from 0.14 to 0.76 on the individual items.

#### 2.2.4 Sleep Diary

The sleep diary was distributed to the parent/caregiver and filled out every day for seven days. The sleep diary enquires about an infant’s sleep duration, sleep onset time, bed time routines (like lullabies, story reading or feeding at bedtime), night wakings, naps and nap routines.

#### 2.2.5 Extracted measures

For comparability across the methods, we extracted four key sleep parameters: day and night sleep duration, and two measures of night waking - night waking number and wake after sleep onset (WASO, the total amount of time a baby spent awake at night). These were selected because they are most commonly used in the literature, therefore allowing for comparison with previous studies. On the sleep questionnaires, day/night sleep duration and WASO are captured by parental estimate of the average duration in the one week prior to the lab visits; night waking number refers to the parent’s estimate of the average number of night wakings in the one week prior to the lab visits. In the diary, parents reported the sleep and wake times every day for a week. Sleep duration / WASO / night waking number is calculated as the 7-day average of the respective measure. We proceeded the same way for actigraphy data, where we used 7-day average data obtained after pre-processing. Completion rates for all sleep measures can be found in Table 1. Pre-processing of actigraph data was done using the ActiLife software v6.3 (ActiGraph Corp.). Raw accelerometer data was aggregated to 60 s epochs. The Sadeh algorithm was used to classify periods of sleeping and waking (Sadeh et al., 1994). This algorithm is the first choice for infant actigraphy data (Meltzer et al., 2012). The Sadeh algorithm is a discriminant function, using only the y-axis information and requiring data in 60 s epochs. The data is segmented into an 11-minute (rolling) window considering the epoch in question as well as the preceding 5 and following 5 epochs. Sadeh’s algorithm has been shown to correctly classify sleep-wake (compared to PSG and compared to observational methods (Sadeh, 1994; Sadeh, Hauri, Kripke, & Lavie, 1995; Sadeh et al., 1994)). Exclusion of non-wear periods was based on the Actilife algorithm and based on parent-report of actigraph removal, i.e., we excluded all periods from analyses where parents indicated that the actigraph was removed. We did not adjust parent-reported bedtimes or wake-up times as we were interested in actigraphy measured timings and how well they coincide with parent-report. We took this decision to make actigraphy a more objective measure that does not rely on parent-report for wake-sleep timings as is sometimes done in the literature (Meltzer et al., 2012).

**Table 1:** Data acquisition rates for each sleep measure (for pooled N).

	**Actigraphy**	**Diary**	**BISQ**	**SSQ**
Data complete	86.1%	84%	98.2%	82.5%
Missing Data	13.9%	16%	1.8%	17.5%

*Note.* BISQ = Brief Infant Sleep Questionnaire, SSQ = Sleep and Settle Questionnaire.

### 2.3 Other measures

#### 2.3.1 Ages & Stages Questionnaire (ASQ)

The ASQ assesses an infant’s activities over time to provide a picture of general developmental status on 5 subscales: communication skills, social skills, problem-solving skills, and fine motor and gross motor skills. The ASQ is a parent-report measure that assesses and infants’ activities over time and provides a picture of general developmental status on five subscales: communication, social personal, problem-solving, fine motor and gross motor. Parents answer the questions with “yes”, “no” and “sometimes”. The questionnaire has high test-retest reliability (94%) and has been validated against another popular assessment of development the Bayley Scales of Infant Development (Schonhaut et al., 2013, Squires et al., 1997). Each age group has their own normed questionnaire which we used for each age group.

#### 2.3.2 Demographics/Socio-economic status (SES)

Parents completed a general questionnaire that enquired about SES status and other demographics, such as highest level of education, years of education, household income, number of bedrooms, parental age and languages spoken at home. Additionally, parents were asked about their medical history including infant birth weight/height, family history of developmental and psychiatric disorders and other circumstances surrounding birth and pregnancy.

#### 2.3.3 Perceived stress Scale (PSS)

The PSS is 10-item questionnaire filled out by the mother to assess life stress. Completion time is around 5 min. A high total score on the PSS is indicative of a larger temporary vulnerability to life-event-evoked depression symptomatology. Predictive validity of PSS is short due to it being influenced by daily events (Cohen, Kamarck, & Mermelstein, 1994).

#### 2.3.4 State trait anxiety inventory (STAI)

The STAI is composed of two sets of 4-point Likert subscales. One set of 20 questions assesses state anxiety (how anxious the person is feeling in a given moment) and one set of 20 questions assesses trait anxiety (a measure of how anxious the person is feeling generally). One key merit is that the questionnaire is easy to understand across different levels of education. Completion times is around 10 min. (Spielberger, 2010).

For data reduction purposes a stress composite score was created from the data of the three stress questionnaires (PSS, STAI-S, STAI-T). The total scores of each questionnaire data set was z-scored and then averaged.

### 2.4 Analysis

Statistical software RStudio Team (2020) with R version 3.5.3 and SPSS v25 (IBM, Chicago, IL) were used for analysing sleep data. For Analysis 1, concordance between different measures (across subjective measures as well as across subjective and objective measures) was assessed using correlational analysis (Pearson's correlation coefficient, intra-class correlation), Bland-Altman plots and equivalence testing using R packages 'blandr' (Datta, 2017) and ‘TOSTER’ (Lakens, Scheel, & Isager, 2018). Descriptive statistics for objective and subjective sleep measures were performed. Mixed model analysis were performed with SPSS v25 (IBM, Chicago, IL) for analysis 3 and 4. Plots were created using Rpackages ‘ggplot2’ and ‘corrplot’ (Wei et al., 2017).

#### 2.4.1 Analysis 1a: Intra-class correlation (ICC)

The ICC is a measure of reliability that can help determine agreement between different raters (in this case the subjective questionnaires and the sleep diary) assessing the same question (in this case sleep parameters). An ICC closer to 0 indicates lower agreement than an ICC closer to 1. A 2-way mixed effects ICC using absolute agreement was used because we aimed to focus on cross-method concordance of the same rater (Fields, 2005; McGraw & Wong, 1996).

#### 2.4.2 Analysis 1b: Bland-Altman plots (BA plots)

Agreement between sleep measures was assessed using Bland-Altman plots as previously (Tikotzky and Volkovich, 2019, Yavuz-Kodat et al., 2019). Limits of agreement are plotted with 95% of the data ideally falling within +/- 1.96 *SD of the mean difference of measures. A line of proportional differences can be drawn and proportional bias towards one measure or the other can be identified by investigating the plots mean difference line (Bland and Altman, 1986, Bland and Altman, 1999).

#### 2.4.3 Analysis 1c: Equivalence test (TOST)

Similarly, to Yavuz-Kodat et al. (2019) equivalence testing is used to identify concordance between measures. First, a range was established under which both methods are considered to be equivalent in terms of the sleep parameter (-δ, δ). This range was established to be 30 min for night sleep duration based on Yavuz-Kodat et al.’s (2019) study, who conducted similar study comparison. However, the range may also be determined by enforcing effect size bounds. The default effect size d = 0.50 was chosen for the other equivalence tests. A significant result for the equivalence test means that the two methods are not showing significantly different means (i.e., can be considered concordant).

#### 2.4.4 Analysis 2: Investigation of factors influencing concordance

In order to investigate the degree to which measure concordance was systematically related to demographic factors, correlational analyses (Pearson’s correlation coefficient, using SPSS v25) between the mean differences of objective and subjective measures and other factors influencing sleep were investigated. These factors included infant age (in days), maternal stress and depression measures and parental demographic information.

#### 2.4.5 Analysis 3 and 4: Relation between sleep and age and cognition

Linear mixed effects models (LMMs) were used to investigate the association between sleep and age and sleep and general development. LMMs were chosen for their ability to handle missing data and for their ability to account for within participant dependence and for inter-individual variability better than comparable analyses like repeated measures ANOVA (Fields, 2005). The accelerated longitudinal design allowed for rapid data collection due to participant’s enrolment at every age. However, this approach meant a higher amount of missing data. Here, a random intercept was added for subject ID and a repeated measures effect for age group. Covariance structures were specified as autoregressive type 1 (AR1) for the repeated group effect, as AR1 covariance structure assumes that two measures taken close together in time from a participant are more likely to be correlated than two measures taken further apart in time (Fields, 2005). Pairwise comparisons corrected for multiple comparisons using the Bonferroni were performed where applicable.

## 3 Results

### 3.1 Descriptive statistics for sleep measures

Table 1 shows data acquisition rates for each sleep measure.

Table 2 shows pooled descriptive statistics across all age groups for sleep questionnaires, actigraphy and sleep diary for the four main sleep parameters (night and day sleep duration and night waking number and WASO).

**Table 2:** Actigraphy-, BISQ-, SSQ-, and diary-measured sleep parameters - Mean and SD for whole sample (all data pooled).

	**Sleep diary** Mean *(SD)*	**Actigraphy**Mean *(SD)*	**BISQ**Mean *(SD)*	**SSQ**Mean (*SD*)
Day sleep duration	133.4 *(43.3)*	115 *(37.9)*	166.6 *(80)*	177.1 *(98.5)*
Night sleep duration	624.3 *(57.3)*	450.9 *(69.8)*	610.5 *(79.8)*	586.5 *(150.5)*
Night waking number	2.2 *(2.1)*	3.9 *(1.2)*	2.3 *(1.7)*	2.4 *(1.8)*
WASO	31.1 *(25.0)*	55.3 *(15.4)*	27.1 *(29.0)*	N/A

*Note.* SD = standard deviation. Durations are in minutes and night waking in number of times awake.

### 3.2 Analysis 1: Concordance between sleep measures

We used Bland-Altman (BA) plots to examine consistency between the objective and the subjective sleep measures respectively (see Fig. 2) and equivalence tests (see SM Section 2) to statistically examine consistency across measures. For detailed statistical results see SM Section 2.

**Fig. 1:**
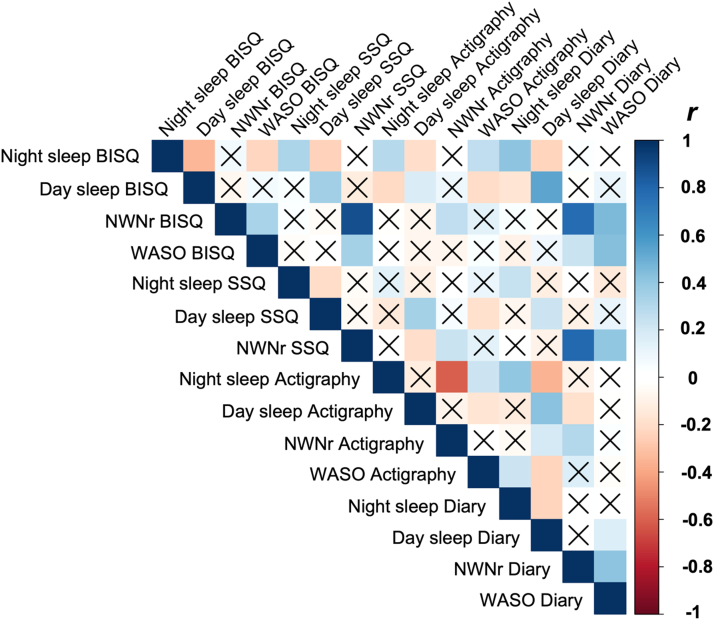
Illustration of correlations between the four different sleep parameters of objective and subjective sleep measures. *Note*. BISQ = Brief Infant Sleep Questionnaire, NWNr = Night waking number, WASO = Wake after sleep onset, SSQ = Sleep & Settle Questionnaire, diary = sleep diary, r = Pearson’s correlation coefficient.

**Fig. 2:**
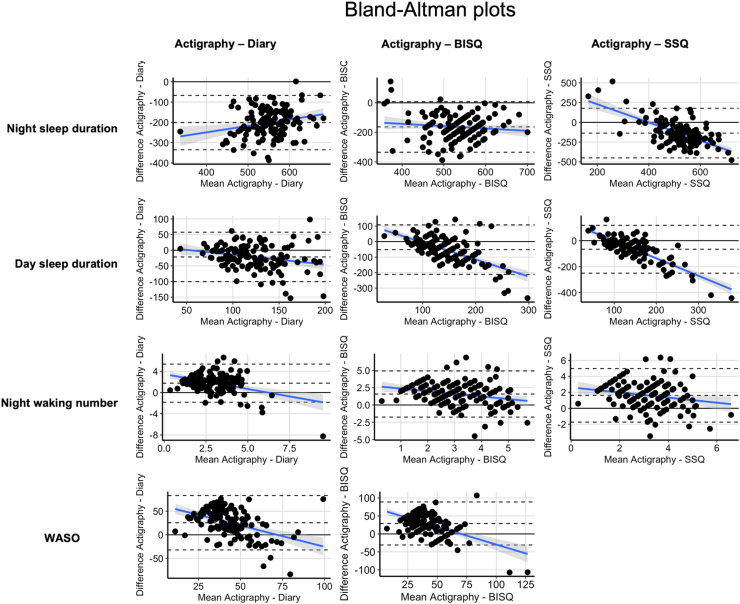
Bland-Altman plots for main sleep parameters. *Note.* Plotting mean of the two measures (x) against the difference of the two measures (y). Blue line = proportional bias line i.e., mean difference between the two measures. Dotted lines = ± 1.96 *SD margins of the mean difference between the measures. If methods show good agreement the bias line should coincide with x = 0 line. If bias line is parallel to x = 0 it means that the bias is the same across participants.

#### 3.2.1 Night sleep duration

**Fig. 3:**
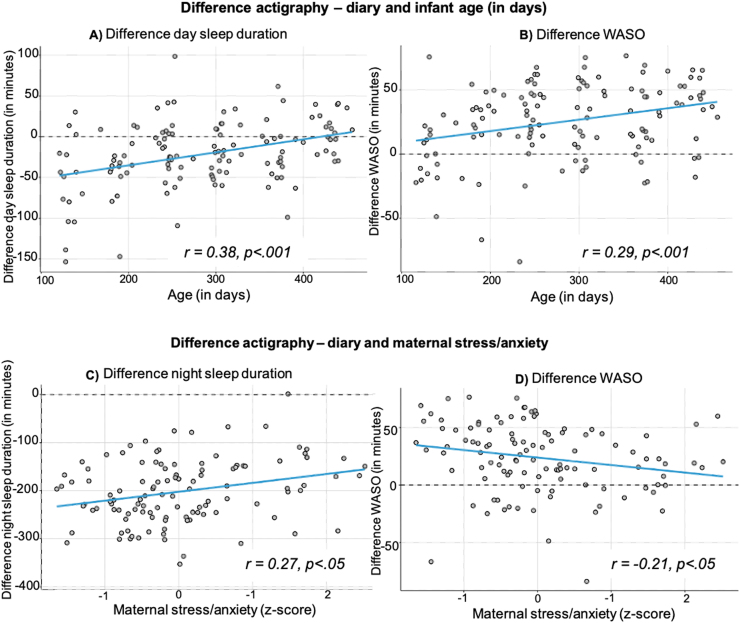
Illustration of cross-method difference of actigraph vs. diary in several sleep variables by infant age and maternal stress/anxiety level. *Notes.* r = Pearson's correlation coefficient, maternal stress/anxiety score = z-scored composite score of PSS and STAI-T/-S.

Raw correlations showed poor to moderate strength of associations between both subjective and objective measures (see Fig. 1). ICC between subjective measures showed poor agreement (see Table 3). BA plots and equivalence analysis showed poor agreement between the different measures. Visual inspection of data suggests presence of a systematic bias (see Fig. 3).

**Table 3:** Intra-class correlation results for subjective sleep parameters.

	**Day sleep duration**	**Night sleep duration**	**Night waking number**
**Single**	.328	.269	.807
**Average**	.594	.525	.926

*Note.* 2-way mixed effects ICC, absolute agreement for BISQ, SSQ, diary. WASO is not included in this analysis as the SSQ does not provide data on wake duration during the night.

#### 3.2.2 Day sleep duration

Raw correlations showed poor to moderate strength of associations between both subjective and objective measures (see Fig. 1). ICC between subjective measures showed poor agreement (see Table 3). BA plots and equivalence analysis showed poor agreement and that most subjective and objective measures were significantly different and not equivalent. BISQ and actigraphy showed a significant equivalence test indicating that the two methods were equivalent [*t*(140) = 1.95, *p* = .03, CI(−24.84;10.27)]. However, the Null Hypothesis Statistical Testing (NHST) was also still significant, indicating that the results of the equivalence test should be interpreted with caution. Visual inspection of data suggests presence of a systematic bias (see Fig. 3).

#### 3.2.3 Night waking number

Raw correlations showed poor to moderate strength of associations between both subjective and objective measures (see Fig. 1). ICC between subjective measures showed good agreement (see Table 3). BA plots and equivalence analysis showed poor agreement and that the subjective and objective measures were significantly different and not equivalent. Visual inspection of data suggests presence of a systematic bias (see Fig. 3).

#### 3.2.4 Wake after sleep onset (WASO)

Raw correlations showed poor to moderate strength of associations between both subjective and objective measures (see Fig. 1). BA plots and equivalence analysis showed poor agreement and that the subjective and objective measures were significantly different and not equivalent. Visual inspection of data suggests presence of a systematic bias (see Fig. 3).

In summary, the different measures did not yield good agreement overall. However, agreement was slightly better for counts of night waking than for duration measures.

### 3.3 Analysis 2: Associations between lack of concordance and maternal and infant characteristics

For analysis 2 we focused on the diary vs. actigraphy comparison in order to reduce the models run. We correlated cross-method agreement across four sleep parameters with infant age, maternal stress score, and parental age. Significant associations (parametric correlations) were found between older infant age and greater consistency (actigraphy vs. diary) in day sleep duration (*r* = 0.38, *p* = <0.001) and WASO (*r* = 0.29, *p* = <0.001) see Fig. 3 for illustration. Mothers who reported being more stressed showed better actigraphy-diary cross-method agreement of night-time sleep parameters like night sleep duration (*r* = 0.27, *p* = .003) and WASO (*r* = −0.21, *p* = .026). Additional analyses showed that older age of mother and father was weakly associated with better actigraphy-diary cross-method agreement of night-time sleep parameters such as night sleep duration (e.g., *r*_*fathers age*_ =0.269, *p* = .004) and WASO (*r*_*fathers age*_ = −0.262, *p* = .006; *r*_*mothers age*_ = −0.192, *p* = .044). Correlation table and additional figures can be found in the SM Section 3.

**Fig. 4:**
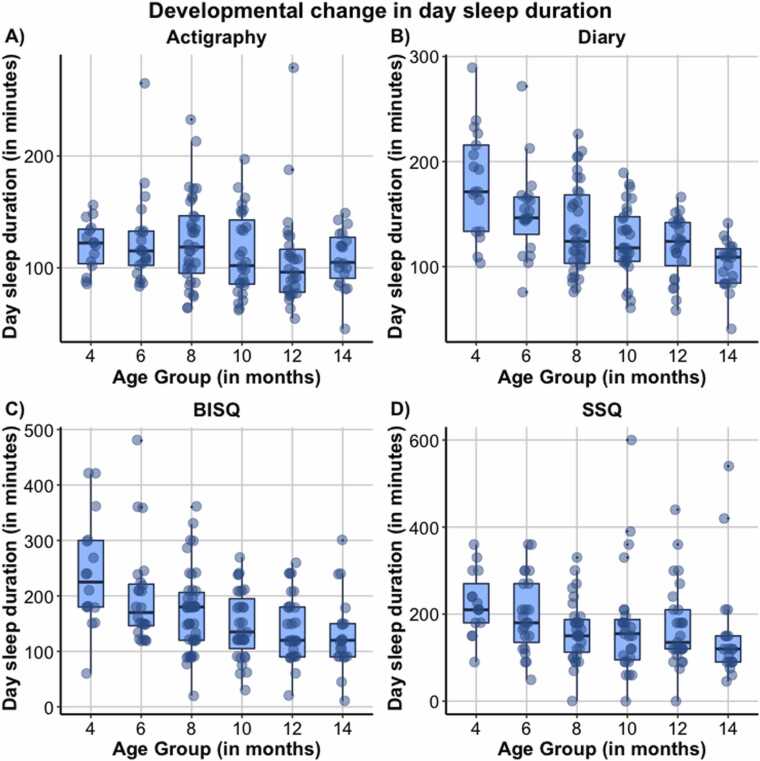
Day sleep duration (in minutes) for Actigraphy (A), Diary (B), Brief Infant sleep questionnaire (BISQ; C) and for Sleep and Settle Questionnaire (SSQ; D). Note. Figures for night waking parameters in SM.

In summary, day sleep was more consistent between actigraphy and diary with increasing age. WASO seems to agree less between actigraphy and diary with increasing age. The difference between actigraphy and diary measures decreased in mothers who reported higher stress for night sleep duration and for WASO.

### 3.4 Analysis 3: Developmental changes in subjective and objective sleep data

**Fig. 5:**
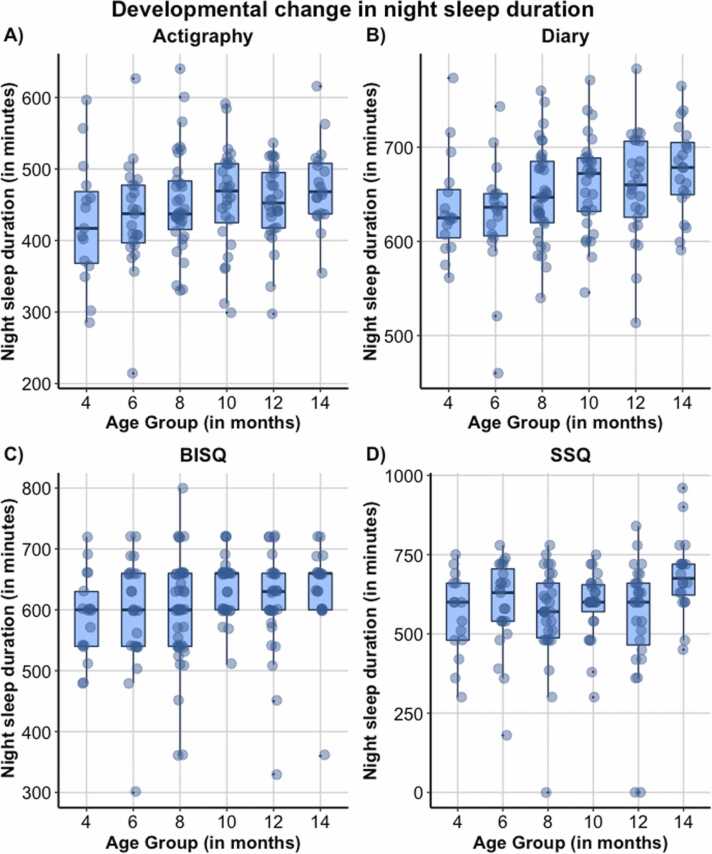
Night sleep duration (in minutes) for Actigraphy (A), Diary (B), Brief Infant sleep questionnaire (BISQ; C) and for Sleep and Settle Questionnaire (SSQ; D). Note. Figures for night waking parameters in SM.

Fig. 5 and Table SM3 in SM Section 4 show developmental trends and full statistics from growth curve models of change in day and night sleep across all measures. Briefly, night waking decreased significantly with age for actigraphy but not parent-report with a linear effect (*F*(1,140) = 8.33, *p* = .005, b = −0.10 ± 0.03). Wake after sleep onset (WASO) increased with age for both actigraphy and BISQ-measured WASO increase with age with a linear (*F*(1,120) = 5.83, *p* = .017, b = 1.01 ± 0.42) and quadratic effect (*F*(1160) = 4.97, *p* = .027, b = 0.53 ± 0.24) respectively; in contrast, diary-measured WASO showed a linear decrease with age (*F*(1,126) = 8.17, *p* = .005, b = −1.97 ± 0.69). Night sleep duration increased linearly with age for both actigraphy (*F*(1,141) = 8.56, *p* = .004, b = 5.63 ± 1.93) and diary (*F*(1,140) = 10.55, *p* = .001, b = 4.69 ± 1.44); but showed a significant cubic effect for the SSQ (*F*(1,128) = 4.00, *p* = .048, b = 0.92 ± 0.46) but not the BISQ. Day sleep duration decreased significantly with age by parent-report but not actigraphy with a linear effect. Taken together, although overall we observed the expected patterns of better quality of sleep with development, there was mixed consistency between different sleep measures in describing patterns of sleep development.

### 3.5 Analysis 4: General infant development (Ages & Stages Questionnaires)

Mean and standard deviations for ASQ subscales and Pearson correlations between the different subscales are reported in SM Section 5.

#### 3.5.1 Night sleep duration

For *objectively measured night sleep* duration only an association with the gross motor subscale was found. There was a significant interaction for age by night sleep duration [*F*(1,138) = 5.00, *p* = .028]. More night sleep was associated with better gross motor skills at younger ages, but with age more night sleep became associated with *poorer* gross motor skills.

For *diary-measured night sleep duration* only an association with the communication subscale was found. There was a significant interaction between age and night sleep duration [*F*(1,88) = 5.23, *p* = .025]. More night sleep was associated with better communication skills in younger infants, but with poorer communication skills in older infants. There were no associations between subjectively or objectively measured night sleep duration and any of the other subscales of ASQ (all ps >0.05; see SM Section 6).

#### 3.5.2 Day sleep duration

**Fig. 6:**
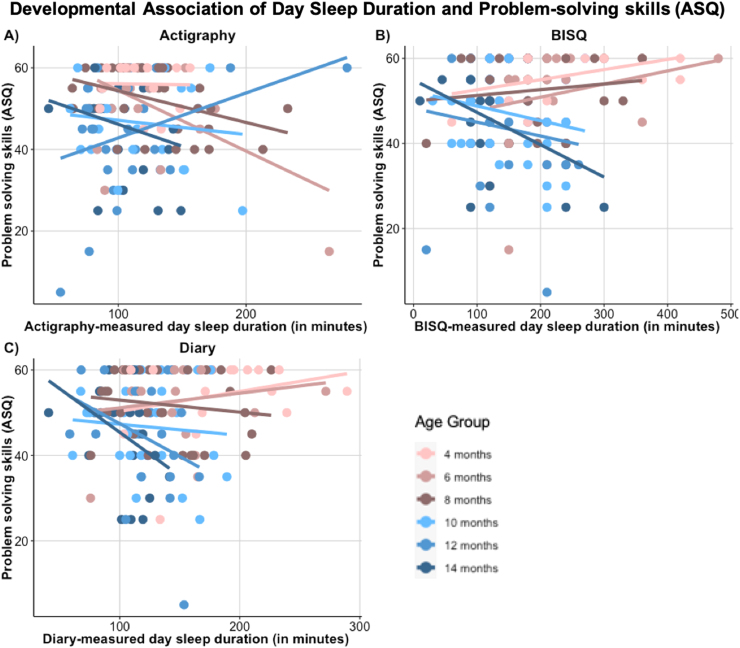
Illustration of the developmental changes in the association between objectively (A) and subjectively (B – C) measured day sleep and parent-reported problem-solving skills. Note. ASQ = Ages & Stages Questionnaire.

**Fig. 7:**
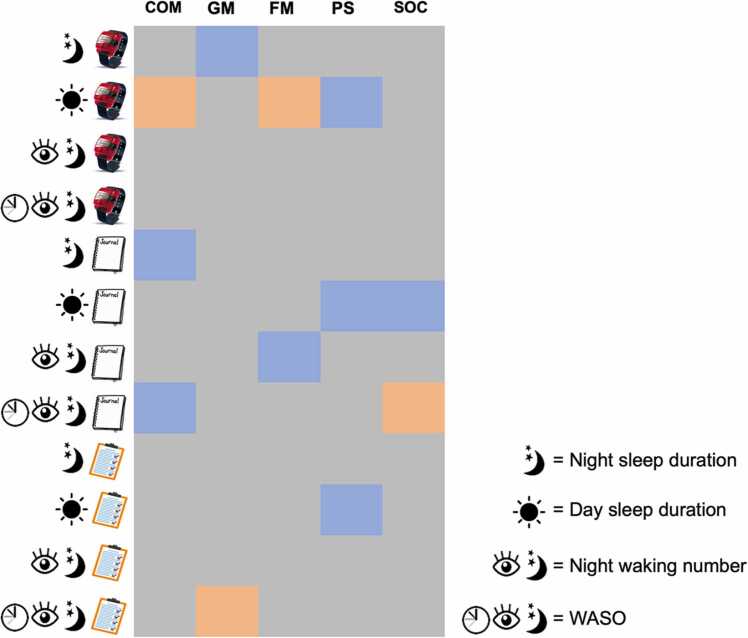
Summary graph of the association of development with sleep parameters. Note. Grey = no association between sleep and development, blue = age-related changes in the association between sleep and development, red = negative association between sleep and development. COM = Communication, GM = Gross motor skills, FM = Fine motor skills, PS = Problem-solving skills, SOC = social skills. For detailed age-related changes see SM and above Section 3.5.

There were significant interaction effects between day sleep duration and age for the problem-solving subscale for the BISQ [*F*(1,148) = 9.28, *p* = .003], diary [*F*(1,131) = 7.77, *p* = .006] and actigraphy [*F*(1,130) = 6.39, *p* = .013]. Longer day sleep duration related to poorer problem-solving and this relation became stronger with age (Fig. 6, Fig. 7). There was a significant interaction between *diary-measured day sleep duration* and social skills [*F*(1,125) = 4.36, *p* = .039]. Longer day sleep related to poorer social skills and this relationship emerged with age. There was also a main effect for *actigraphy-measured day sleep duration* [*F*(1,133) = 4.71, *p* = .032, b = −0.092 ± 0.035, *t*(121) = −2.61] on communication subscale, with lower communication scores associating with more day sleep. Finally there was a main effect for *actigraphy-measured day sleep duration* [*F*(1,134) = 5.66, *p* = .019, b = −0.054 ± 0.023, *t*(134) = −2.38] on fine motor subscale, with lower fine motor scores relating to more day sleep. Thus, more day sleep appeared to be associated with lower developmental skills across a range of methods.

#### 3.5.3 Night waking number

There was a significant interaction between *diary-measured night waking number* [*F*(1,98) = 7.80, *p* = .006] and age on the fine motor subscales of the ASQ. More night wakings were associated with lower fine motor skills in younger infants, but stronger fine motor skills in older infants. There were no associations with *objectively measured* or *BISQ-measured night waking number* and any of the subscales of the ASQ.

#### 3.5.4 WASO

There was a significant interaction between *diary-measured WASO* and age [*F*(1,127) = 4.14, *p* = .044] on communication subscales of the ASQ. Visual inspection showed more WASO was associated with better communication skills in younger infants, but poorer communication skills in older infants. There was a main effect of *diary-measured WASO* on the social subscale [*F*(1,131) = 3.90, *p* = .05, b = −0.079 ± 0.040, *t*(131) = −1.98], with lower social skills associating with more WASO. There was also a main effect of *BISQ-measured WASO* on the gross motor subscale [*F*(1,134) = 3.95, *p* = .049], with lower gross motor skills relating to more WASO. Finally, there were no associations with *objectively measured WASO* and any of the subscales of the ASQ.

#### 3.5.5 Summary

Results from the mixed model analysis showed that day sleep duration showed cross-method-consistent developmental changes in the association with the problem-solving ASQ subscale. More day sleep in general seemed to be associated with poorer parent-reported general development. Findings regarding other parameters were more fragmented, though many parameters showed age-related changes in the association of sleep and general development (Fig. 7).

## 4 Discussion

In this accelerated longitudinal study of infants aged between 4 and 14 months, we measured both parent-report and actigraphy measures of sleep for a week and collected parent-report measures of infant development and parental characteristics such as maternal stress. Different sleep measures showed overall low concordance. Correlational analyses and linear mixed modelling also showed that concordance between objective and subjective measures differed with age and maternal stress, and different sleep measures showed different relations to cognition. Taken together, these results suggest a highly nuanced view of the relation between infant habitual sleep and development is warranted.

### 4.1 Agreement of sleep measures

Overall, subjective measures (sleep diary and questionnaires) did not yield good agreement with each other, or with the objective measure (actigraphy) across most sleep parameters. Night waking parameters yielded slightly better cross-method agreement than sleep duration assessments, though some discrepancies remained. This is likely because it might be easier for parents to remember how often their child woke up than to remember the actual duration of the sleep. These cross-method disparities have several implications for research and the conclusions that sleep researchers draw about sleep parameters. It might be prudent to treat studies that find effects with regard to sleep duration with caution, especially if only parent-report measures are used. Moreover, sleep parameters and method choice need to be carefully deliberated and rooted in theoretical reasoning. For instance, it may be possible that parental perception of infant sleep is particularly interesting to the overarching research question, in which case subjective measures should be chosen. In addition, direct cross-study comparisons should be undertaken only if similar methods were used (i.e., either objective or subjective).

### 4.2 Influence of confounding variables on agreement between sleep measures

Cross-method agreement was related to infant age as well as maternal stress and anxiety levels. For example, mothers reporting higher stress and anxiety levels and mothers with younger infants showed better cross-method agreement on night waking parameters. These results illustrate the importance of a holistic view of studying sleep and development that considers information about the broader family context, similarly to theories proposed by El-Sheikh, Sadeh and colleagues (El-Sheikh and Sadeh, 2015, Sadeh et al., 2010). Interestingly, it was the cross-method agreement that was particularly affected; follow-up analyses generally did not reveal a direct association between objectively or subjectively measured infant sleep and stress measures. This could imply that rather than directly impacting infant sleep, stress is more related to the degree to which parents are aware of their infant’s sleep patterns.

It could be that stressed parents are they themselves more awake at night (Taylor, Lichstein, Durrence, Reidel, & Bush, 2005) and therefore more aware of their infant’s sleep pattern or that they spend more time being concerned about their child’s development/sleep consequently paying more attention to their infant’s sleep. This also highlights possible reasons for cross-method disparities and underscores the need to carefully consider method choice in conducting and evaluating (developmental) sleep studies.

Further, our work implies that studies using heterogenous samples to study the relation between maternal stress and infant sleep could inadvertently introduce bias if relying on parent-report measures of sleep. This may also be relevant to developmental studies that investigate sleep differences between typically developing and infants with neurodevelopmental disorders (as done in MacDuffie et al., 2020) since there is evidence of higher parental stress in children with neurodevelopmental disorders (Hayes & Watson, 2013). This could mean, for example, that sleep reporting is more consistent in the high stress group (i.e., parents of children with neurodevelopmental disorders), resulting in potential group differences reported in sleep patterns when perhaps there is no underlying difference. Further, developmental changes in parent-report of infant sleep may interact with temperament differences in infants: for example, for infants who learn self-soothing more rapidly, parents may report less night waking (Goodlin-Jones, Burnham, Gaylor, & Anders, 2001). Research reporting associations between infant sleep and temperament could support this (Morales-Muñoz et al., 2020, Spruyt et al., 2008). Researchers might conclude the presence of age-related changes when in practice these only reflect the degree in which the child’s behaviour is noticed by parents.

### 4.3 Considering infant sleep measurement choice for developmental research

**Fig. 8:**
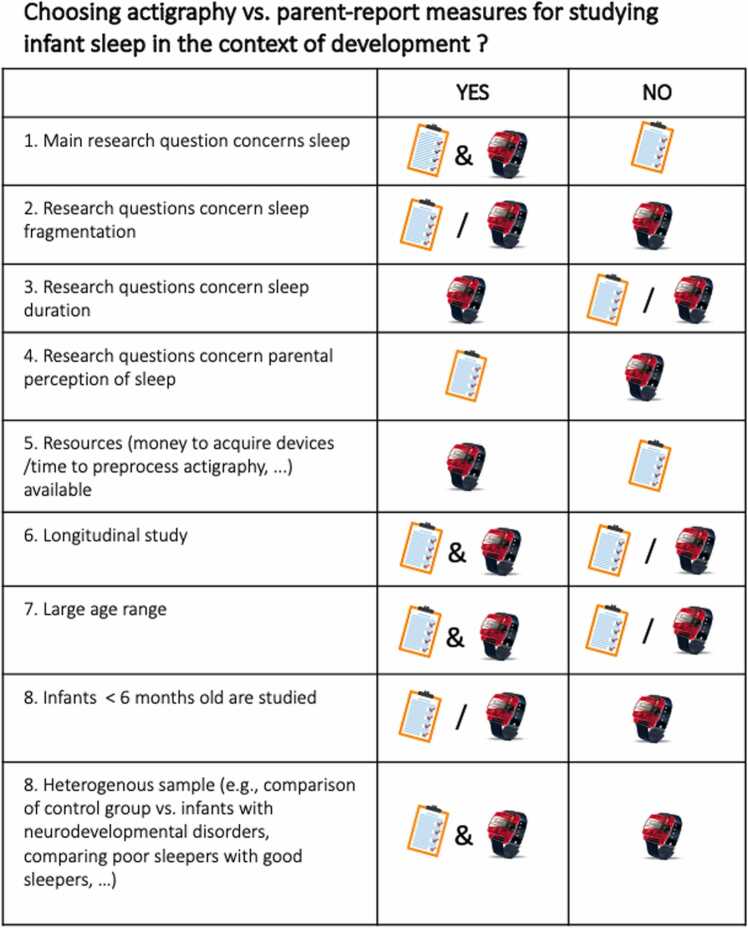
Illustration of questions to consider when choosing actigraphy vs. parent-report measures. Note. & = both actigraph and questionnaires can be chosen. / = either actigraph or parent-report can be chosen.

Both actigraphy and parent-report have advantages and disadvantages; thus, no definite recommendation can be made with regard to which method is “better”. However, Fig. 8. represents an attempt to aid researchers in choosing a sleep measure for their study. Researchers selecting a sleep assessment method need to consider the following factors. First, the research question needs to be considered. For example, if parental perception of sleep or the effects of sleep on the family are of interest, parent-report may be more appropriate than actigraphy. Considering the investigation of sleep problems, if parents do not notice their child waking up, it likely has less impact on family functioning than children that do wake up their parents, in which case parent-report may be very valuable.

Secondly, available resources need to be considered. Actigraphy devices are more expensive than parent-report measures and pre-processing of actigraphy data is time-intensive (Meltzer et al., 2012). Other considerations centre around the sample and study design. For example, if the sample is likely to be heterogenous in terms of parental characteristics (e.g., comparing different SES) both parent-report and actigraphy should be used to account for cross-method differences driven by parental characteristics. Similarly, in longitudinal studies testing infants many times or in studies testing large age ranges, multiple measures should be used. In homogenous samples and narrow age groups this is likely less of an issue, as sleeps varies less (Bathory and Tomopoulos, 2017, Gorgoni et al., 2019). Lastly, for populations with unusual movement patterns (e.g., cerebral palsy) or infants who mainly sleep in buggies or car seats, actigraphy might be less reflective of true, underlying sleep (Meltzer et al., 2012, Galland et al., 2018, Hutson and Snow, 2020).

### 4.4 Influence of methodological choice on associations with age and with general development

The above results pose the question of which method to use when assessing sleep in relation to development. When investigating developmental changes in sleeping patterns in the sample, the sample largely showed results in line with prior literature (Peters, 2017, Gorgoni et al., 2019). A reduction in day sleep duration, an increase in night sleep duration and a general decline in night waking number was found across measures. However, objective and subjective measures showed inconsistent patterns of change with age. For example, while actigraphy-measured night sleep was associated with gross motor skills (such as in Pisch, 2015) the same pattern was not found in the diary- or BISQ-measured data. This finding emphasises how cross-method differences might influence conclusions about change in infant sleep with age, similar to Horger et al.’s (2021b) conclusion. It is possible that developmental milestones, such as onset of crawling as suggested by Scher and Cohen (2015) impact infants' sleep quality but that these changes are captured by only one of methods.

Further, inconsistencies between sleep assessment methods also emerged in the association with parent-reported general infant development (as assessed by the Ages & Stages Questionnaire subscales). Parent-report questionnaires offer a straightforward method to understand an infant’s development and to capture it across a broad range of contexts. This also has the advantage of being less affected by infant state at a one-time lab observation. However, results showed that the association between ASQ subscales and measures of night sleep duration or night waking parameters did not converge across methods. No associations with development were found with objectively measured night waking parameters. Subjectively measured night waking parameters did not relate consistently to development across the two parameters that are closely linked (WASO and night waking number) or across the diary and questionnaire measures. As we have shown, consistency between sleep measures in the sample was not high especially with regard to duration measures, perhaps explaining why the diary and questionnaires were also inconsistently related to measures of development. However, the lack of consistent association with the ASQ for diary-measured WASO and night waking number is particularly puzzling as prior research shows that higher night waking number is associated with higher WASO values (Bathory & Tomopoulos, 2017). This may indicate that associations between development and only one sleep measure are likely to be spurious, and caution should be taken when drawing conclusions. It may also be possible that specific patterns are picked up by one method but not by the other.

Of note our developmental measurements were parent-report and therefore could have shared measure variance consequently correlating more closely with parent-report measures of sleep. However, convergent evidence of a relation between day sleep duration and problem-solving skills indicates that this is a promising avenue for future investigation.

### 4.5 The importance of studying day sleep in association with infant development

More notable are the consistent findings regarding day sleep duration across both objective and subjective sleep measures. To summarise our findings, higher parent-reported day sleep was associated with lower scores on problem-solving skills, especially in older infants. Objectively measured sleep showed the same pattern for fine motor skills. Higher objectively measured day sleep duration was also associated with lower parent-reported communication skills. Communication skills were also associated with diary-measured day sleep duration, though there were age-related changes not consistent with the actigraphy findings. BISQ findings were non-significant. In addition, there were also age-related changes in the association between day sleep duration and social skills with older but not younger infants having lower social skills the more they slept. Below we explain these results and contextualise them against the background of current literature.

In our study day sleep duration seems to be associated with aspects of infant development, and in particular with parent-reported problem-solving skills. This is in line with recent studies that have recognised the importance of studying habitual day sleep (Horváth & Plunkett, 2018). Day sleep has thus far mainly been studied to identify sleep microstructural compositions in a nap and to study the benefit of sleeping on prior learning rather than investigating habitual day sleep patterns in infants specifically (e.g., Friedrich et al., 2017, Friedrich et al., 2018, Friedrich et al., 2020; Horváth, Hannon, Ujma, Gombos, & Plunkett, 2017; Horváth, Hannon, Ujma, Gombos, & Plunkett, 2018; Konrad, Seehagen, Schneider, & Herbert, 2016; Seehagen, Konrad, Herbert, & Schneider, 2015). Research into habitual day sleep has shown that more parent-reported day time naps at 7 months predicts vocabulary growth in early childhood (Horváth & Plunkett, 2016). Infants whose parents indicated on the BISQ that they napped frequently were better at generalising previously memorised information (Lukowski & Milojevich, 2013). Further, Berger and Scher (2017) showed that infants who napped were actually better at a motor problem-solving task (Berger & Scher, 2017). However, contrary to these prior results that generally highlight a benefit of napping for development, our results suggest that higher day sleep was associated with lower parent-reported problem-solving skills and fine motor skills.

One possibility is that while daytime napping is beneficial for immediate learning, there is a dose-response relationship such that too much daytime sleep is detrimental to general development as suggested also in prior research (Könen et al., 2015, Kocevska et al., 2017, Horger et al., 2021a). It is possible that day sleep hinders development of problem-solving skills perhaps by way of giving infants comparably less time to practice their skills. Indeed, Bernier et al. (2010) found that one year old infants who sleep more during the night than during the day relative to their peers have better expressive vocabulary skills 14 months later (Bernier, Carlson, Bordeleau, & Carrier, 2010). Similarly, Kocevska et al. (2017) found an inverted U relationship of sleep duration in toddlerhood and cognitive measures in childhood where both too much and too little sleep were detrimental. Of course, this research is somewhat contrary to the long-standing evidence in the field that show benefits of daytime napping for processes that benefit general development such as memory consolidation (e.g., Friedrich et al., 2020; Kurdziel, Duclos, & Spencer, 2013; Werchan, Kim, & Gómez, 2021). Future research should aim to integrate both streams of research.

Conversely, it is also possible that infants who are more advanced in their development sleep less during day and have better problem-solving skills, both changes associated with increased age. Infants who develop better problem-solving skills may not need to consolidate as much information during the day, thereby sleeping less. Infants with inherent delays in development could exhibit differences in day sleeps / their sleep cycle consolidation as a symptom, in the same way in which younger infants sleep more during the day. This is supported by the age-related changes such that associations between more day sleep and poorer developmental skills were more pronounced in older infants. Lastly, it is possible that day sleep is predominantly influenced by parental background, such as education. For example, at 6 months, infants of mothers with lower education levels showed higher day sleep duration than infants of mothers with higher education levels (Yu et al., 2020). While we could not actively control for it due to power issues, simple correlations and descriptive statistics did not show differences across maternal educational background and sleep parameters in the present sample. Overall, our work indicates that the influence and meaning of day sleep is an important area for future investigation, particularly when considering implications for public policy such as napping practices in day care settings. For example, it is possible that napping in older infants and toddlers might not always be beneficial.

### 4.6 Limitations and future directions

One limitation is that we did not have a ‘ground truth’ measure of true underlying infant sleep to which our objective and subjective habitual sleep measures may be compared. Utilising PSG measurement in-home in infants in a large-scale longitudinal study is not currently feasible, though this may become possible with better technology in the future, such as wireless one-channel EEG (Lucey et al., 2016). Furthermore, the sample size precluded the use of more complex statistical approaches such as structural equation modelling, cascade analyses or path analysis that would be sensitive to developmental ordering; future research should focus on exploring the causal nature of those associations further. Another limitation is that our assessment of general development was based on parent-report of infant skill level. While this approach was appropriate in order to compare this study’s results to prior research that employed such measures (e.g., Pisch, 2015), parent-report is potentially biased as discussed in 1.2, 1.3 and 2.2. of this manuscript and in de Los Reyes and Kazdin (2005). It might be important to investigate the role of day sleep on general development as assessed by a researcher, such as via the Mullen Scales of Early Learning (Mullen, 1995). Future research should therefore aim to incorporate such measures into the study design. Moreover, reporting accuracy for day and night sleep is likely affected by different factors, e.g., night sleep might be more accurate for infants that wake their parents whereas daytime for infants that are better settlers. Another limitation of the current study is that we were not able to consider infant feeding method as suggested by Rudzik et al. (2018). Our extensive study protocol included questions about feeding method, however there was a large percentage of missing data for the feeding method questions, and therefore this data could not be included in the analyses. Missing data could have been due to the large number of questions parents were asked to fill out in the protocol resulting in some questions not being answered. Our future work might benefit from redesigning the questions surrounding infant feeding method, by making them more succinct. However, based on Rudzik et al. (2018) feeding method can impact concordance between objective and subjective measures, so underlying differences in feeding method might have contributed further to the differences in cross-method agreement observed here. Future studies should therefore aim to disentangle the bias in cross-method agreement further and investigate influences beyond infant age and maternal stress, such as infant mood /temperament, comprehensive measures of socioeconomic status (e.g., neighbourhood deprivation) or infant feeding method.

### 4.7 Conclusion

In conclusion, our study found that subjective and objective sleep assessment methods showed mixed agreement. Cross-method agreement was also associated with maternal stress levels and infant age. Objective and subjective measures did not yield the same patterns of association with developmental domains, indicating that sleep method choice can influence which associations are found between sleep and cognitive development. Moreover, our findings that day sleep might not be as beneficial in older infants suggests further investigation is needed that centres around the benefits and detriments of habitual day sleep in development. These results have wide-range implications for sleep method choice in developmental studies.

## Funding

This work was supported by the European Union's HORIZON 2020 Research and Innovation Programme under the Marie Sklodowska-Curie Grant Agreement No 721895 (United Kingdom) and an ISSF2 Wellcome Trust postdoctoral fellowship Grant Agreement No 204770/Z/16/Z (United Kingdom).

## CRediT authorship contribution statement

**Louisa K. Gossé**: Conceptualization, Data collection, Formal analysis, Writing - original draft; Writing - review & editing; **Frank Wiesemann:** Conceptualization, Funding acquisition, Writing - review & editing; **Clare Elwell:** Conceptualization; **Emily J.H. Jones**: Conceptualization, Funding acquisition, Writing - review & editing.
