# Supplementary material for: Concordance between subjective and objective measures of infant sleep varies by age and maternal mood: Implications for studies of sleep and cognitive development
Source: Infant Behav Dev. 2022 Feb;66:101663. doi: 10.1016/j.infbeh.2021.101663 (PMC8803548; doi:10.1016/j.infbeh.2021.101663)
Supplement: Supplementary file 1 — Supplementary material. [file mmc1.docx]

**Supplementary material**

**Section 1 - Instructions to parents regarding sleep measurements**


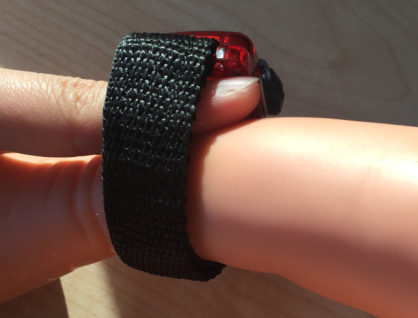
An actigraph is a small wearable, wireless device that measures activity patterns (see pictures) and can be attached either to your baby’s ankle or wrist. It is widely used in sleep research and also in infant research. It has been approved and deemed safe by experts for use in baby and toddler studies. We are asking you to put the actigraphy device on your baby for a period of 7 days.

This monitor is easily fit around your baby’s ankle with the actigraph facing towards the outside. The size of the band of the monitor can be easily adjusted as it is a soft velcro strap that you can close and adjust yourself to make it comfortable for your baby. Usually we recommend leaving a finger-wide amount of space between the actigraph and the skin when you attach it (see right). You can put your babies sock over the actigraph to cover it if you want to. We will show you exactly the safest manner to attach the activity watch to your baby’s ankle in person during your first visit at the Babylab and you will be able to practice under supervision. If you decide to remove the activity monitor at some point, you can do so the same way you are attaching the monitor: simply by opening the velcro strap and sliding the actigraph over your baby’s foot. We just ask you to record the time and the day when you decide to remove it.

We are doing this as we are interested in how your baby’s sleep changes during the night and during the day. Please return the device when you come back to the Babylab for your second visit as we will need it to read the data. As mentioned above, despite numerous quality assurance and device safety approvals, because a baby’s skin is so sensitive it could be that their skin becomes irritated or that a rash develops. Should you notice this, remove the activity monitor and contact us immediately (point of contact below).

**How to use the Actigraph**

You are being asked to place the actigraph on your baby’s ankle for 7 days in order to measure his/her sleep. We are interested in both night-time sleep, as well as daytime sleep, so please keep the activity device on all day and all night during these 7 days.

Here is how to put the actigraph together:

1. Put the strap through the opening on the side of the actigraph
2. Pull the strap through the plastic buckle and then put the strap through the opening on the other side of the actigraph. Please make sure the strap is straight and not twisted.
3. Pull the strap down and stick the velcro together to secure the actigraph.

Place the actigrpah on your baby’s ankle. Please note in your Sleep Diary on which ankle you have placed the device (left or right). After you have placed the actigraph on your baby, please ensure that there is enough space between your baby’s ankle and the band. To make sure it’s comfortable for your baby, you should be able to place a finger in between his/her ankle and the band.

After the device is on, please put a sock or long pijamas over the actigraph, and check again that the sock or pijama leg is not too tight in the area where the actigraph is. This will help ensure that the device stays in place.

**Sleep Diary Information**

1. Over the course of 7 days, while your baby is wearing the actigraph, please fill out the sleep diary that has been given to you.
2. Please answer the questions approximately at the same time each day (e.g. always mornings, …)
3. Please try to make note of the exact times pertaining to the questions in the diary about your baby’s sleep (e.g. when did your go to sleep, when did your baby wake up, etc.)
4. It may be helpful for you to write down your baby’s exact sleep times in the moment, so that you don’t have to try to remember it until the time that you fill out the questions for the day

**If you have any questions pertaining to answering the questions in the sleep diary, please do not hesitate to contact us immediately as we will be more than happy to help!**

**Section 2 - Equivalence tests**

Equivalence tests were performed to study whether actigraphy and diary were truly not equivalent as suggested by inspection of the BA plots. For all sleep variables, actigraphy measures were significantly different/ not equivalent from diary measures. Illustration of results for main sleep parameters for actigraphy vs. diary (all p’s <.001 for NHST and all p’s >.10 for TOST) can be seen below (*Figure SM1*) and detailed statistical test results for actigraphy vs. BISQ / actigraphy vs. diary can be found below in *Table SM1*. Of note, for Day Sleep Duration BISQ and actigraphy showed a significant equivalence test indicating that the two methods were equivalent [*t*(140) = 1.95, *p* = .03, CI(-24.84;10.27)]. However, the NHST was also still significant, indicating that the results of the equivalence test should be interpreted with caution.

**
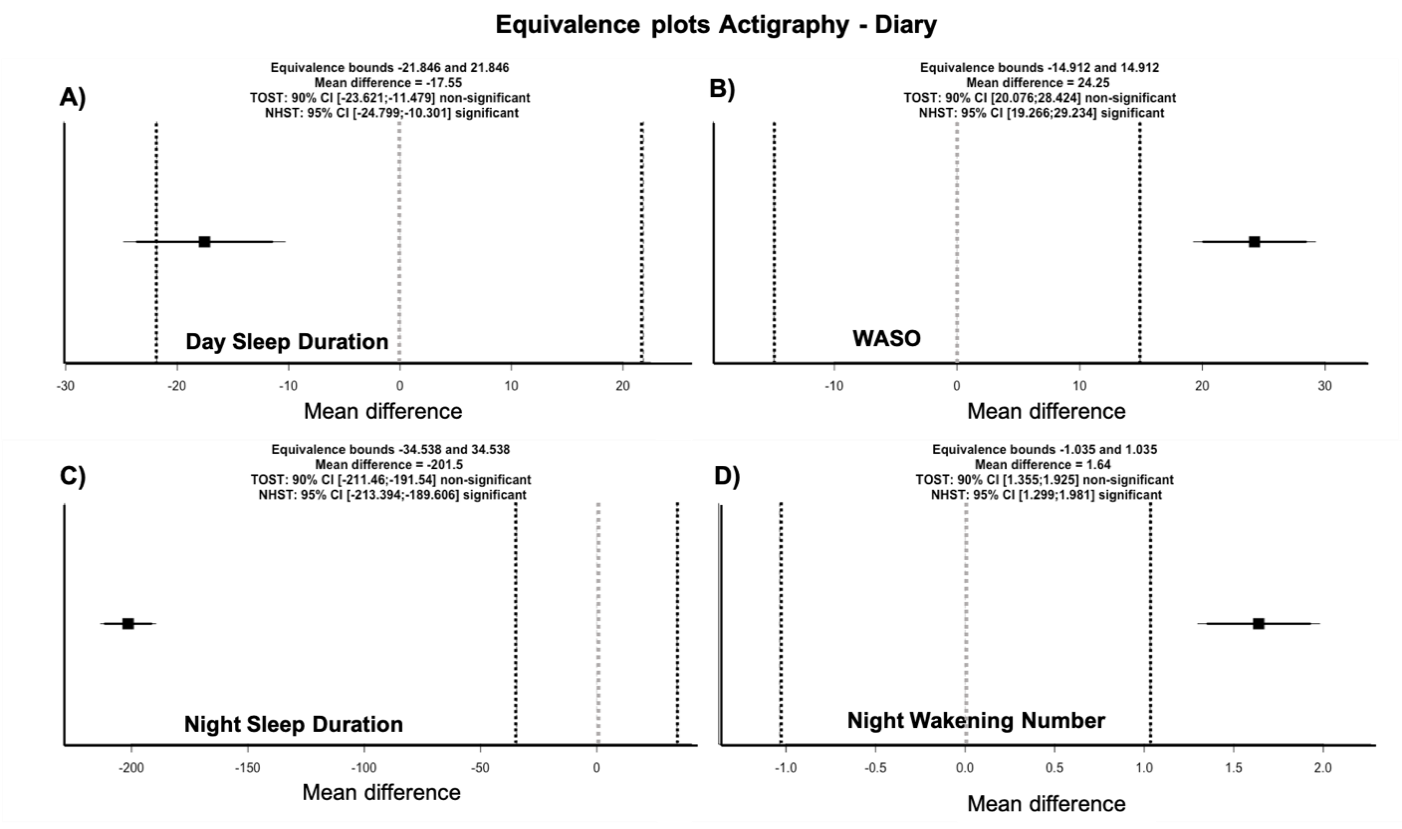
**

*Figure SM1.* Equivalence plots for main sleep parameters (A) Day Sleep Duration, B) WASO, C) Night Sleep Duration, D) Night Waking Number. *Note.* NHST = Null Hypothesis Testing, TOST = equivalence test, CI = confidence interval (plots produced with R package ‘TOSTER’)

| *Table SM1.* Results of the Equivalence testing for actigraphy vs diary and actigraphy vs BISQ | | | | | | | | |
| --- | --- | --- | --- | --- | --- | --- | --- | --- |
| **Actigraphy vs diary** | | | | | | | | |
|  |  |  | **NHST** | | | **TOST** | | |
|  | **Raw Equiva-lence bounds** | **df** | **t** | **p** | **95 % CI** | **t** | **p** | **90 % CI** |
| Day Sleep Duration | ± 21.85 | 141 | -4.79 | <.001 | [-24.80;  -11.48] | 1.17 | .12 | [-23.85;  -11.48] |
| Night Sleep Duration | ± 34.54 | 131 | -33.51 | <.001 | [-213.39;  -189.61] | -27.77 | 1.00 | [-211.46;  -191.54] |
| WASO | ± 14.91 | 139 | 9.26 | <.001 | [19.27; 29.23] | 3.71 | 1.00 | [20.08;  28.42] |
| Night Waking Number | ± 1.04 | 143 | 9.51 | <.001 | [1.30;  1.98] | 3.51 | 1.00 | [1.36;  1.93] |
| **Actigraphy vs BISQ** | | | | | | | | |
|  |  |  | **NHST** | | | **TOST** | | |
|  | Raw Equi-valence bounds | df | t | p | 95 % CI | t | p | 90 % CI |
| Day Sleep duration | ± 26.12 | 140 | -3.99 | <.001 | [-26.25;  -8.86] | 1.95 | .03* | [-24.84;  -10.27] |
| Night Sleep Duration | ± 44.36 | 145 | -21.84 | <.001 | [172.52;148.21] | -15.80 | 1.00 | [-174.87;  -145.85] |
| WASO | ±16.28 | 148 | 10.586 | <.001 | [22.97;  33.51] | 4.483 | 1.00 | [23.82;  32.66] |
| Night Waking Number | ± 0.92 | 146 | 10.29 | <.001 | [1.26;  1.86] | 4.29 | 1.00 | [1.31  ;1.81] |
| *Notes.* CI = confidence interval; df = degrees of freedom, NHST = Null Hypothesis testing, TOST = test of significance | | | | | | | | |

**Section 3 - Concordance analysis**

We checked individual differences in concordance of some participants to assess whether parental description of infant sleep patterns coincided better with actigraphy. This was done for the actigraphy vs. diary data only. See *Figure SM2* for details.

*Table SM2* shows correlation table of cross-method agreement of sleep parameters (WASO, night and day sleep duration, night waking number) with parental factors and infant age.


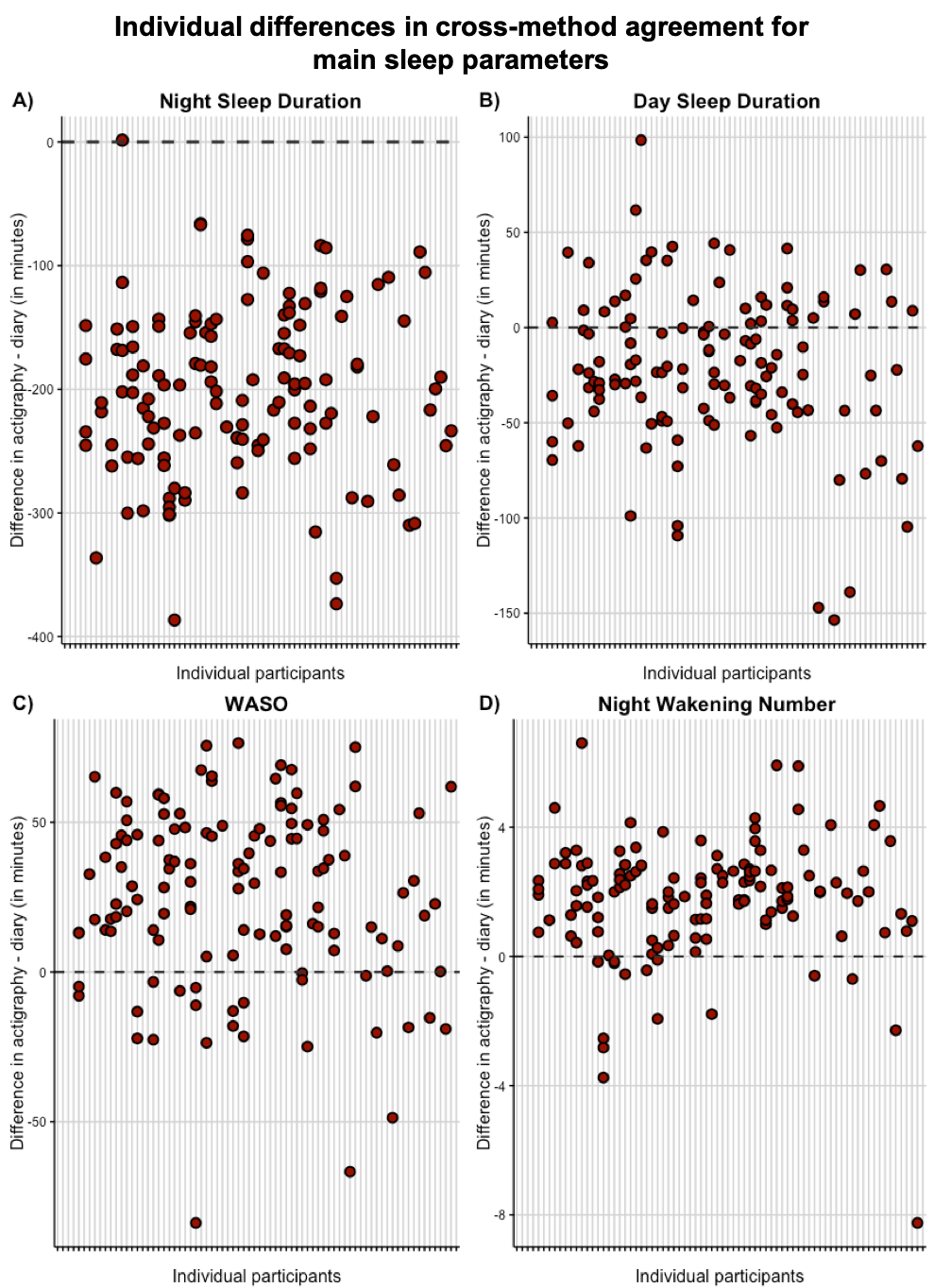


*Figures SM2.* Illustration showing the mean differences (actigraphy - diary, y-axis) per participant (x-axis) in Night Sleep Duration (A), Day Sleep Duration (B), WASO (C) and Night Waking Number (D). Dotted line indicates perfect agreement of actigraphy and diary. The further the individual scatter dot is from the vertical line the worse the parent-reported sleep variable estimation compared to actigraphy.

| *Table SM2.* Correlations cross-method agreement, parental factors and infant age. | | | | | | | | |
| --- | --- | --- | --- | --- | --- | --- | --- | --- |
|  |  | Difference Day sleep duration | Difference WASO | Difference Night waking number | Maternal stress score | Mothers age | Fathers age | Infant age |
| Difference Night sleep duration | Correlation | .195 | -.007 | -.265 | .275 | .169 | .217 | .001 |
|  | *p-value* | *.028* | *.937* | *.002* | *.003* | *.193* | *.096* | *.987* |
| Difference Day sleep duration | Correlation |  | .199 | .012 | .006 | .044 | -.096 | .378 |
|  | *p-value* |  | *.027* | *.898* | *.952* | *.737* | *.471* | *.000* |
| Difference WASO | Correlation |  |  | .235 | -.211 | -.07 | -.218 | .294 |
|  | *p-value* |  |  | *.007* | *.026* | *.596* | *.097* | *.001* |
| Difference Night waking number | Correlation |  |  |  | -.039 | -.048 | .009 | .090 |
|  | *p-value* |  |  |  | *.676* | *.714* | *.917* | *.304* |
| Maternal stress score | Correlation |  |  |  |  | -.065 | .080 | .009 |
|  | *p-value* |  |  |  |  | *.612* | *.535* | *.917* |
| Mothers age | Correlation |  |  |  |  |  | .644 | -.067 |
|  | *p-value* |  |  |  |  |  | *.000* | *.582* |
| Fathers age | Correlation |  |  |  |  |  |  | -.115 |
|  | *p-value* |  |  |  |  |  |  | *.343* |
|  | | | | | | | | |

**Section 4 - Developmental changes in sleep parameters**

*Figure SM3* shows developmental changes in night waking number and WASO and *Table SM3* shows Growth Curve Models for different sleep measures and parameters


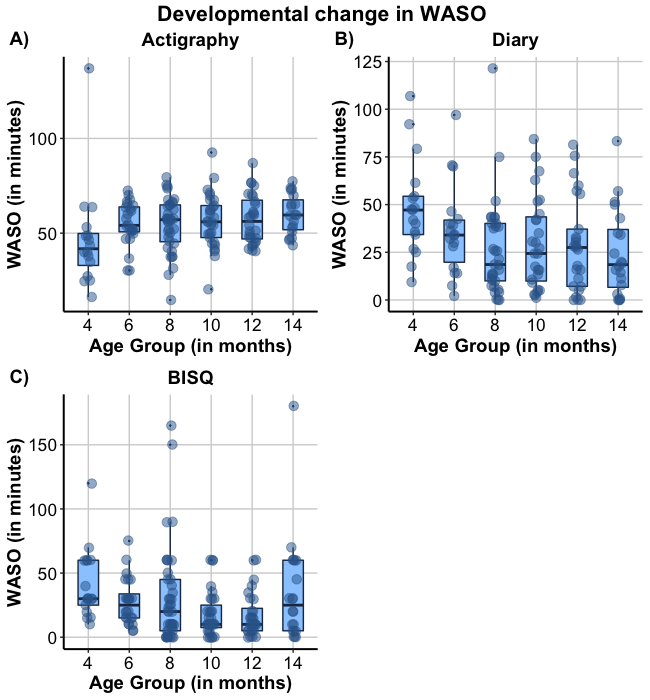


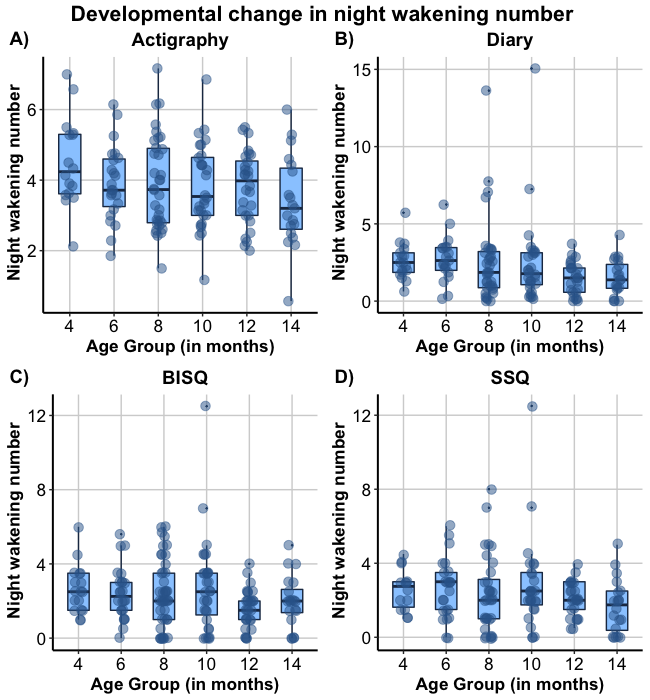


*Figure SM3* a) Developmental change in night waking number b) Developmental change in WASO.

| *Table SM3.*  Results of Growth Curve Models for different sleep measures and parameters | | | | | | | |
| --- | --- | --- | --- | --- | --- | --- | --- |
|  | **Parameter** | **Effect** | **F** | **df** | **p** | **b** | **SE** |
| Night sleep duration | Actigraphy | Linear | 8.56 | 141 | .004** | 5.63 | 1.93 |
|  | Diary | Linear | 10.55 | 140 | .001*** | 4.69 | 1.44 |
|  | BISQ | - | 2.83 | 140 | .095 | 3.68 | 2.19 |
|  | SSQ | Cubic | 4.00 | 128 | .048* | 0.92 | 0.46 |
| Day sleep duration | Actigraphy |  | 2.26 | 118 | .136 | -1.67 | 1.11 |
|  | Diary | Linear | 43.70 | 125 | <.001*** | -7.17 | 1.08 |
|  | BISQ | Linear | 23.11 | 134 | <.001*** | -9.92 | 2.06 |
|  | SSQ | Linear | 4.05 | 122 | .046* | -5.44 | 2.70 |
| Night waking number | Actigraphy | Linear | 8.33 | 140 | .005** | -0.10 | 0.03 |
|  | Diary | - | 2.65 | 144 | .106 | -0.09 | 0.06 |
|  | BISQ | - | 1.31 | 157 | .253 | -0.05 | 0.05 |
|  | SSQ | - | 1.08 | 136 | .300 | -0.05 | 0.05 |
| WASO | Actigraphy | Linear | 5.83 | 120 | .017* | 1.01 | 0.42 |
|  | Diary | Linear | 8.17 | 126 | .005** | -1.97 | 0.69 |
|  | BISQ | Quadratic | 4.97 | 160 | .027* | 0.53 | 0.24 |
| *Notes.* WASO = Wake after sleep onset; SE = Standard Error, * p < .05, ** p <.01, ***p < .001. | | | | | | | |

**Section 5 - Ages and Stages in association with sleep parameters**

*Table SM4* and *Tables SM5* show descriptive statistics and correlations of the different subscales of the Ages and Stages Questionnaire.

| *Table SM4.* Descriptive Statistics ASQ | | | | | | |
| --- | --- | --- | --- | --- | --- | --- |
|  | | ASQ: COM | ASQ: GM | ASQ: FM | ASQ: PS | ASQ: SOC |
| Mean | 47.16 | | 47.50 | 50.28 | 48.85 | 46.53 |
| SD | 10.99 | | 13.65 | 10.19 | 11.09 | 11.63 |
| *Notes.* ASQ = Ages and Stages Questionnaire; COM = communication subscale; GM = gross motor subscale, FM = fine motor subscale; PS = problem-solving subscale; SOC= social subscale. | | | | | | |

|  | | | | | | | | | | | |  |
| --- | --- | --- | --- | --- | --- | --- | --- | --- | --- | --- | --- | --- |
| *Table SM5.* Correlations amongst different subscales of the Ages and Stages Questionnaire. | | | | | | | | | | | |  |
|  | |  | | ASQ: GM | | ASQ: FM | | ASQ: PS | | ASQ: SOC | | |
| ASQ: COM | Correlation | | .228 | | .340 | | .388 | | .347 | |  |  |
|  | *p-value* | | *.004* | | *.000* | | *.000* | | *.000* | |  |  |
| ASQ: GM | Correlation | |  | | .130 | | .106 | | .307 | |  |  |
|  | *p-value* | |  | | *.099* | | *.182* | | *.000* | |  |  |
| ASQ: FM | Correlation | |  | |  | | .439 | | .363 | |  |  |
|  | *p-value* | |  | |  | | *.000* | | .*000* | |  |  |
| ASQ:  PS | Correlation | |  | |  | |  | | .415 | |  |  |
|  | *p-value* | |  | |  | |  | | *.000* | |  |  |
| *Note.* ASQ = Ages and Stages Questionnaire; COM = communication subscale; GM = gross motor subscale, FM = fine motor subscale; PS = problem-solving subscale; SOC= social subscale | | | | | | | | | | |  |  |

**Section 6 - Linear mixed models Ages and Stages Questionnaire – sleep parameters**

*Table SM6a-e* reports the statistical results and model fit statistics for the Linear mixed models run in Analysis 4.

| *Table SM6a.* Linear mixed model statistics for the association of ASQ and sleep parameters of three methods (Actigraphy, diary, BISQ) – Communication subscale | | | | |
| --- | --- | --- | --- | --- |
|  | **-2LL** | **AIC** | **BIC** | **df** |
| **Sleep measure: Actigraphy** | | | | |
| **ASQ: Communication subscale** | | | | |
| Night sleep duration | | | | |
| M1 | 1088.16 | 1100.16 | 1117.98 | 6 |
|  | Age: *F*(1,94) = 5.99, *p* = .016  Night sleep duration: *F*(1,120) = 0.34, *p* = .56 | | | |
| M2 | 1086.02 | 1100.02 | 1120.81 | 7 |
|  | Age: *F*(1,138) = 1.61, *p* = .28  Night sleep duration: *F*(1,130) = 1.43, *p* = .23  Age x night sleep duration: *F*(1,136) = 2.24, *p* = .14 | | | |
| Day sleep duration | | | | |
| M1 | 1049.11 | 1061.11 | 1078.72 | 6 |
|  | Age: *F*(1,95) = 9.26, *p* = .003  Day sleep duration: *F*(1,136) = 2.41, *p* = .123 | | | |
| M2 | 1045.89 | 1059.89 | 1080.43 | 7 |
|  | Age: *F*(1,138) = 1.61, *p* = .28  Day sleep duration: *F*(1,130) = 1.43, *p* = .23  Age x day sleep duration: *F*(1,136) = 2.24, *p* = .14 | | | |
| WASO |  |  |  |  |
| M1 | 1120.30 | 1132.30 | 1150.33 | 6 |
|  | Age: *F*(1,94) = 8.25, *p* = .005  WASO: *F*(1,147) = 1.20, *p* = .276 | | | |
| M2 | 1120.22 | 1134.22 | 1155.25 | 7 |
|  | Age: *F*(1,138) = 1.23, *p* = .259  WASO: *F*(1,141) = 0.03, *p* = .858  Age x WASO: *F*(1,133) = 0.08, *p* = .772 | | | |
| Night waking number | | | | |
| M1 | 1094.05 | 1106.05 | 1123.91 | 6 |
|  | Age: *F*(1,97) = 5.83, *p* = .018  WASO: *F*(1,123) = 1.11, *p* = .295 | | | |
| M2 | 1093.62 | 1107.62 | 1128.56 | 7 |
|  | Age: *F*(1,114) = 2.18, *p* = .142  WASO: *F*(1,126) = 0.11, *p* = .746  Age x WASO: *F*(1,123) = 0.54, *p* = .465 | | | |
| **Sleep measure: Diary** | | | | |
| **ASQ: Communication subscale** | | | | |
| Night sleep duration | | | | |
| M1 | 1047.21 | 1059.21 | 1076.91 | 6 |
|  | Age: *F*(1,94) = 10.41, *p* = .002  Day sleep duration: *F*(1,1116) = 0.18, *p* = .67 | | | |
| M2 | 1042.53 | 1056.53 | 1077.17 | 7 |
|  | Age: *F*(1,88) = 3.99, *p* = .049  Day sleep duration: *F*(1,99) = 3.60, *p* = .06  Age x day sleep duration: *F*(1,88) = 5.23, *p* = .025 | | | |
| Day sleep duration |  |  |  |  |
| M1 | 1040.83 | 1052.83 | 1070.48 | 6 |
|  | Age: *F*(1,99) = 7.58, *p* = .007  Day sleep duration: *F*(1,132) = 0.24, *p* = .627 | | | |
| M2 | 1040.83 | 1054.83 | 1075.42 | 7 |
|  | Age: *F*(1,99) = 7.58, *p* = .007  Day sleep duration: *F*(1,132) = 0.24, *p* = .627  Age x day sleep duration: *F*(1,121) = 0.001, *p* = .98 | | | |
| WASO |  |  |  |  |
| M1 | 1018.91 | 1030.91 | 1048.43 | 6 |
|  | Age: *F*(1,90) = 14.06, *p* < .001  WASO: *F*(1,130) = 1.76 *p* = .187 | | | |
| M2 | 1015.02 | 1029.02 | 1049.41 | 7 |
|  | Age: *F*(1,106) = 0.67, *p* = .415  WASO: *F*(1,123) = 1.91, *p* = .169  Age x WASO: *F*(1,127) = 4.14, *p* = .044 | | | |
| Night waking number |  |  |  |  |
| M1 | 1045.83 | 1057.83 | 1075.52 | 6 |
|  | Age: *F*(1,96) = 11.00, *p* = .001  Night waking number: *F*(1,122) = 0.76, *p* = .386 | | | |
| M2 | 1045.71 | 1059.71 | 1080.35 | 7 |
|  | Age: *F*(1,98) = 2.51, *p* = .116  Night waking number: *F*(1,114) = 0.29, *p* = .589  Age x Night waking number: *F*(1,107) = 0.12, *p* = .727 | | | |
| **Sleep measure: BISQ** |  |  |  |  |
| **ASQ: Communication subscale** | | | | |
| Night sleep duration |  |  |  |  |
| M1 | 1200.44 | 1212.44 | 1239.93 | 6 |
|  | Age: *F*(1,96) = 6.34, *p* = .013  Day sleep duration: *F*(1,159) = 3.65, *p* = .058 | | | |
| M2 | 1197.98 | 1211.98 | 1233.55 | 7 |
|  | Age: *F*(1,152) = 1.60, *p* = .208  Day sleep duration: *F*(1,145) = 0.81, *p* = .370  Age x day sleep duration: *F*(1,151) = 2.59, *p* = .109 | | | |
| Day sleep duration |  |  |  |  |
| M1 | 1197.00 | 1209.00 | 1227.45 | 6 |
|  | Age: *F*(1,101) = 5.47, *p* = .021  Day sleep duration: *F*(1,156) = 0.47, *p* = .505 | | | |
| M2 | 1197.00 | 1211.00 | 1232.51 | 7 |
|  | Age: *F*(1,121) = 1.01, *p* = .316  Day sleep duration: *F*(1,145) = 0.13, *p* = .715  Age x day sleep duration: *F*(1,141) = 0.16, *p* = .899 | | | |
| WASO |  |  |  |  |
| M1 | 1183.30 | 1195.30 | 1213.71 | 6 |
|  | Age: *F*(1,100) = 7.02, *p* = .009  Day sleep duration: *F*(1,136) = 0.69, *p* = .409 | | | |
| M2 | 1182.71 | 1196.71 | 1218.19 | 7 |
|  | Age: *F*(1,123) = 1.67, *p* = .199  Day sleep duration: *F*(1,132) = 2.12, *p* = .148  Age x day sleep duration: *F*(1,125) = 4.36, *p* = .039 | | | |
| Night waking number |  |  |  |  |
| M1 | 1204.01 | 1216.01 | 1234.50 | 6 |
|  | Age: *F*(1,93) = 7.63, *p* = .007  Night waking number: *F*(1,138) = 0.01, *p* = .940 | | | |
| M2 | 1204.00 | 1218.00 | 1239.55 | 7 |
|  | Age: *F*(1,95) = 2.91, *p* = .091  Night waking number: *F*(1,130) = 0.03, *p* = .876  Age x Night waking number: *F*(1,127) = 0.03, *p* = .854 | | | |

| *Table SM6b.* Linear mixed model statistics for the association of ASQ and sleep parameters of three methods (Actigraphy, diary, BISQ) – Gross motor subscale | | | | |
| --- | --- | --- | --- | --- |
|  | **-2LL** | **AIC** | **BIC** | **df** |
| **Sleep measure: Actigraphy** | | | | |
| **ASQ: Gross motor subscale** | | | | |
| Night sleep duration | | | | |
| M1 | 1151.10 | 1163.10 | 1180.92 | 6 |
|  | Age: *F*(192) = 2.32, *p* = .132  Day sleep duration: *F*(1,125) = 0.30, *p* = .58 | | | |
| M2 | 1146.25 | 1160.25 | 1181.03 | 7 |
|  | Age: *F*(1,139) = 6.03, *p* = .015  Day sleep duration: *F*(1,129) = 3.50, *p* = .064  Age x day sleep duration: *F*(1,138) = 5.00, *p* = .028 | | | |
| Day sleep duration |  |  |  |  |
| M1 | 1112.97 | 1124.97 | 1142.58 | 6 |
|  | Age: *F*(1,93) = 1.14, *p* = .288  Day sleep duration: *F*(1,135) = 1.08, *p* = .301 | | | |
| M2 | 1112.91 | 1126.91 | 1147.45 | 7 |
|  | Age: *F*(1,124) = 0.00, *p* = .956  Day sleep duration: *F*(1,132) = 0.29, *p* = .590  Age x day sleep duration: *F*(1,129) = 0.07, *p* = .786 | | | |
| WASO | | | | |
| M1 | 1187.49 | 1199.49 | 1217.51 | 6 |
|  | Age: *F*(1,95) = 2.52, *p* = .116  WASO: *F*(1,132) = 0.97, *p* = .325 | | | |
| M2 | 1185.87 | 1199.87 | 1220.89 | 7 |
|  | Age: *F*(1,135) = 3.05, *p* = .083  WASO: *F*(1,133) = 0.59, *p* = .446  Age x WASO: *F*(1,126) = 1.68, *p* = .198 | | | |
| Night waking number | | | |  |
| M1 | 1155.72 | 1167.72 | 1185.58 | 6 |
|  | Age: *F*(1,87) = 3.51, *p* = .064  Night waking number: *F*(1,129) = 3.95, *p* = .049 | | | |
| M2 | 1154.96 | 1168.96 | 1189.80 | 7 |
|  | Age: *F*(1,110) = 0.06, *p* = .808  Night waking number: *F*(1,124) = 0.02, *p* = .891  Age x Night waking number: *F*(1,121) = 0.78, *p* = .378 | | | |
| **Sleep measure: Diary** | | | |  |
| **ASQ: Gross motor subscale** | | | | |
| Night sleep duration |  |  |  |  |
| M1 | 1120.24 | 1132.24 | 1149.94 | 6 |
|  | Age: *F*(1,99) = 7.58, *p* = .600  Day sleep duration: *F*(1,132) = 0.24, *p* = .166 | | | |
| M2 | 1117.96 | 1131.96 | 1152.60 | 7 |
|  | Age: *F*(1,99) = 2.44, *p* = .122  Day sleep duration: *F*(1,105) = 2.73, *p* = .056  Age x day sleep duration: *F*(1,95) = 2.31, *p* = .13 | | | |
| Day sleep duration |  |  |  |  |
| M1 | 1110.77 | 1122.77 | 1140.42 | 6 |
|  | Age: *F*(1,87) = 1.36, *p* = .25  Day sleep duration: *F*(1,138) = 0.25, *p* = .870 | | | |
| M2 | 1109.36 | 1123.36 | 1143.95 | 7 |
|  | Age: *F*(1,96) = 2.40, *p* = .12  Day sleep duration: *F*(1,120) = 1.13, *p* = .253  Age x day sleep duration: *F*(1,107) = 1.43, *p* = .235 | | | |
| WASO |  |  |  |  |
| M1 | 1088.58 | 1100.58 | 1118.10 |  |
|  | Age: *F*(1,81) = 0.63, *p* = .43  WASO: *F*(1,132) = 0.02, *p* = .882 | | | |
| M2 | 1086.63 | 1100.63 | 1121.07 |  |
|  | Age: *F*(1,95) = 2.62, *p* = .109  WASO: *F*(1,113) = 1.88, *p* = .173  Age x WASO: *F*(1,119) = 1.99, *p* = .161 | | | |
| Night waking number | | | | |
| M1 | 1119.97 | 1131.97 | 1149.66 |  |
|  | Age: *F*(1,84) = 1.43, *p* = .236  Night waking number: *F*(1,124) = 2.14, *p* = .146 | | | |
| M2 | 1119.91 | 1133.91 | 1154.55 |  |
|  | Age: *F*(1,89) = 0.24, *p* = .625  Night waking number: *F*(1,109) = 0.01, *p* = .905  Age x Night waking number: *F*(1,100) = 0.05, *p* = .817 | | | |
| **Sleep measure: BISQ** | | | | |
| **ASQ: Gross Motor subscale** | | | | |
| Night sleep duration |  |  |  |  |
| M1 | 1278.54 | 1290.54 | 1309.03 | 6 |
|  | Age: *F*(1,101) = 1.60, *p* = .208  Night sleep duration: *F*(1,160) = 2.67, *p* = .104 | | | |
| M2 | 1278.33 | 1292.33 | 1313.90 | 7 |
|  | Age: *F*(1,152) = 0.08, *p* = .78  Night sleep duration: *F*(1,150) = 0.01, *p* = .922  Age x Night sleep duration: *F*(1,152) = 0.21, *p* = .647 | | | |
| Day sleep duration |  |  |  |  |
| M1 | 1272.82 | 1284.82 | 1303.27 | 6 |
|  | Age: *F*(1,109) = 2.49, *p* = .117  Day sleep duration: *F*(1,160) = 0.18, *p* = .673 | | | |
| M2 | 1272.75 | 1286.75 | 1308.28 | 7 |
|  | Age: *F*(1,132) = 1.00, *p* = .322  Day sleep duration: *F*(1,150) = 0.16, *p* = .692  Age x day sleep duration: *F*(1,150) = 0.07, *p* = .794 | | | |
| WASO | | | | |
| M1 | 12.64.74 | 1276.74 | 1295.15 | 6 |
|  | Age: *F*(1,101) = 2.45, *p* = .121  WASO: *F*(1,153) = 0.39, *p* = .535 | | | |
| M2 | 1261.28 | 1275.28 | 1296.76 | 7 |
|  | Age: *F*(1,104) = 6.18, *p* = .014  WASO: *F*(1,134) = 3.95, *p* = .049  Age x WASO: *F*(1,129) = 3.56, *p* = .0361 | | | |
| Night waking number | | | | |
| M1 | 1279.48 | 1291.48 | 1310.00 | 6 |
|  | Age: *F*(1,97) = 2.84, *p* = .095  Night waking number: *F*(1,141) = 1.73, *p* = .190 | | | |
| M2 | 1279.32 | 1293.32 | 1314.89 | 7 |
|  | Age: *F*(1,96) = 0.38, *p* = .165  Night waking number: *F*(1,119) = 0.20, *p* = .653  Age x Night waking number: *F*(1,108) = 0.61, *p* = .436 | | | |

| *Table SM6c.* Linear mixed model statistics for the association of ASQ and sleep parameters of three methods (Actigraphy, diary, BISQ) – Fine motor subscale | | | | |
| --- | --- | --- | --- | --- |
|  | **-2LL** | **AIC** | **BIC** | **df** |
| **Sleep measure: Actigraphy** | | | | |
| **ASQ: Fine motor subscale** | | | | |
| Night sleep duration |  |  |  |  |
| M1 | 1065.09 | 1077.09 | 1094.91 | 6 |
|  | Age: *F*(1,76) = 0.21, *p* = .65  Day sleep duration: *F*(1,119) = 0.01, *p* = .921 | | | |
| M2 | 1064.24 | 1078.24 | 1099.03 | 7 |
|  | Age: *F*(1,130) = 0.98, *p* = .325  Day sleep duration: *F*(1,125) = 0.67, *p* = .413  Age x day sleep duration: *F*(1,126) = 0.87, *p* = .35 | | | |
| Day sleep duration |  |  |  |  |
| M1 | 1023.97 | 1035.97 | 1053.57 | 6 |
|  | Age: *F*(1,77) = 0.05, *p* = .82  Day sleep duration: *F*(1,134) = 5.66, *p* = .019 | | | |
| M2 | 1022.55 | 1036.55 | 1057.09 | 7 |
|  | Age: *F*(1,113) = 1.54, *p* = .217  Day sleep duration: *F*(1,131) = 3.36, *p* = .069  Age x day sleep duration: *F*(1,125) = 1.49, *p* = .224 | | | |
| WASO |  |  |  |  |
| M1 | 1097.15 | 1109.15 | 1127.17 |  |
|  | Age: *F*(1,76) = 0.08, *p* = .78  WASO: *F*(1,142) = 0.00, *p* = .959 | | | |
| M2 | 1096.90 | 1110.90 | 1131.93 | 7 |
|  | Age: *F*(1,115) = 0.32, *p* = .575  WASO: *F*(1,129) = 0.23, *p* = .629  Age x WASO: *F*(1,108) = 0.26, *p* = .614 | | | |
| Night waking number |  |  |  |  |
| M1 | 1070.33 | 1082.33 | 1100.19 | 6 |
|  | Age: *F*(1,76) = 0.37, *p* = .55  Night waking number: *F*(1,110) = 0.97, *p* = .33 | | | |
| M2 | 1068.12 | 1082.12 | 1102.96 | 7 |
|  | Age: *F*(1,82) = 1.57, *p* = .214  Night waking number: *F*(1,103) = 1.09, *p* = .298  Age x Night waking number: *F*(1,92) = 2.30, *p* = .133 | | | |
| Sleep measure: Diary |  |  |  |  |
| ASQ Fine motor subscale |  |  |  |  |
| Night sleep duration |  |  |  |  |
| M1 | 1033.19 | 1045.19 | 1062.88 |  |
|  | Age: *F*(1,82) = 0.17, *p* = .683  Day sleep duration: *F*(1,114) = 0.38, *p* = .537 | | | |
| M2 | 1030.96 | 1044.96 | 1065.60 |  |
|  | Age: *F*(1,88) = 2.38, *p* = .126  Day sleep duration: *F*(1,103) = 1.35, *p* = .247  Age x day sleep duration: *F*(1,89) = 2.30, *p* = .133 | | | |
| Day sleep duration |  |  |  |  |
| M1 | 1025.20 | 1037.20 | 1054.84 | 6 |
|  | Age: *F*(1,83) = 0.41, *p* = .52  Day sleep duration: *F*(1,120) = 1.13, *p* = .253  Age x day sleep duration: *F*(1,107) = 1.43, *p* = .235 | | | |
| M2 | 1025.13 | 1039.13 | 1059.72 | 7 |
|  | Age: *F*(1,91) = 0.00, *p* = .992  Day sleep duration: *F*(1,116) = 0.16 *p* = .694  Age x day sleep duration: *F*(1,101) = 0.07, *p* = .790 | | | |
| WASO |  |  |  |  |
| M1 | 1000.08 | 1012.08 | 1029.60 |  |
|  | Age: *F*(1,74) = 0.12, *p* = .726  WASO: *F*(1,121) = 2.97, *p* = .09 | | | |
| M2 | 998.24 | 1012.24 | 1032.68 | 7 |
|  | Age: *F*(1,90) = 1.61, *p* = .207  WASO: *F*(1,105) = 0.01, *p* = .057  Age x WASO: *F*(1,110) = 1.91, *p* = .17 | | | |
| Night waking number |  |  |  |  |
| M1 | 1033.94 | 1045.94 | 1063.64 |  |
|  | Age: *F*(1,82) = 0.08, *p* = .780  Night waking number: *F*(1,117) = 0.61, *p* = .435 | | | |
| M2 | 1026.42 | 1040.42 | 1061.06 |  |
|  | Age: *F*(1,88) = 4.46, *p* = .038  Night waking number: *F*(1,108) = 6.30, *p* = .014  Age x Night waking number: *F*(1,98) = 7.80, *p* = .006 | | | |
| **Sleep measure: BISQ** |  |  |  |  |
| **ASQ: fine motor subscale** |  |  |  |  |
| Night sleep duration |  |  |  |  |
| M1 | 1186.84 | 1198.84 | 1217.33 |  |
|  | Age: *F*(1,85) = 0.34, *p* = .56  Night sleep duration: *F*(1,158) = 0.01, *p* = .91 | | | |
| M2 | 1186.61 | 1200.61 | 1222.18 |  |
|  | Age: *F*(1,151) = 0.30, *p* = .58  Night sleep duration: *F*(1,147) = 0.17, *p* = .68  Age x Night sleep duration: *F*(1,150) = 0.23, *p* = .63 | | | |
| Day sleep duration |  |  |  |  |
| M1 | 1180.19 | 1192.19 | 1210.64 | 6 |
|  | Age: *F*(1,87) = 0.18, *p* = .68  Day sleep duration: *F*(1,132) = 0.16 *p* = .63 | | | |
| M2 | 1179.63 | 1193.63 | 1215.16 | 7 |
|  | Age: *F*(1,101) = 0.74, *p* = .391  Day sleep duration: *F*(1,130) = 0.28, *p* = .601  Age x day sleep duration: *F*(1,120) = 0.57, *p* = .452 | | | |
| WASO |  |  |  |  |
| M1 | 1173.00 | 1185.00 | 1203.41 |  |
|  | Age: *F*(1,88) = 0.18, *p* = .671  WASO: *F*(1,117) = 0.69, *p* = .51 | | | |
| M2 | 1171.42 | 1185.42 | 1206.90 |  |
|  | Age: *F*(1,82) = 0.29, *p* = .591  Day sleep duration: *F*(1,100) = 2.08, *p* = .152  Age x day sleep duration: *F*(1,88) = 1.64, *p* = .203 | | | |
| Night waking number |  |  |  |  |
| M1 | 1186.55 | 1198.55 | 1217.04 |  |
|  | Age: *F*(1,80) = 0.39, *p* = .535  Night waking number: *F*(1,119) = 0.31, *p* = .578 | | | |
| M2 | 1184.51 | 1198.51 | 1220.08 |  |
|  | Age: *F*(1,98) = 0.68, *p* = .412  Night waking number: *F*(1,110) = 1.50, *p* = .224  Age x Night waking number: *F*(1,107) = 2.11, *p* = .149 | | | |

| *Table SM6d.* Linear mixed model statistics for the association of ASQ and sleep parameters of three methods (Actigraphy, diary, BISQ) – Problem-solving subscale | | | | |
| --- | --- | --- | --- | --- |
|  | **-2LL** | **AIC** | **BIC** | **df** |
| **Sleep measure Actigraphy** | | | | |
| **ASQ: Problem-solving subscale** |  |  |  |  |
| Night sleep duration |  |  |  |  |
| M1 | 1079.45 | 1091.45 | 1109.26 | 6 |
|  | Age: *F*(1,106) = 13.99, *p* < .001  Day sleep duration: *F*(1,118) = 0.44, *p* = .508 | | | |
| M2 | 1092.66 | 1092.66 | 1113.45 | 7 |
|  | Age: *F*(1,139) = 0.09, *p* = .760  Day sleep duration: *F*(1,130) = 0.37, *p* = .544  Age x day sleep duration: *F*(1,138) = 0.83, *p* = .365 | | | |
| Day sleep duration |  |  |  |  |
| M1 | 1046.41 | 1058.41 | 1076.02 | 6 |
|  | Age: *F*(1,107) =14.84, *p* < .001  Day sleep duration: *F*(1,138) = 0.55, *p* = .461 | | | |
| M2 | 1040.19 | 1054.19 | 1074.73 | 7 |
|  | Age: *F*(1,127) = 12.53, *p* = .001  Day sleep duration: *F*(1,135) = 7.02, *p* = .009  Age x day sleep duration: *F*(1,130) = 6.39, *p* = .013 | | | |
| WASO |  |  |  |  |
| M1 | 1116.13 | 1128.13 | 1146.11 |  |
|  | Age: *F*(1,111) = 13.62, *p* < .001  WASO: *F*(1,147) = 1.10 *p* = .297 | | | |
| M2 | 1115.83 | 1129.83 | 1150.81 |  |
|  | Age: *F*(1,138) = 0.32, *p* = .576  WASO: *F*(1,141) = 0.86, *p* = .354  Age x WASO: *F*(1,133) = 0.30, *p* = .583 | | | |
| Night waking number |  |  |  |  |
| M1 | 1096.35 | 1108.35 | 1126.21 | 6 |
|  | Age: *F*(1,106) = 11.20, *p* = .001  Night waking number: *F*(1,121) = 0.09, *p* = .766 | | | |
| M2 | 1095.79 | 1109.79 | 1130.62 | 7 |
|  | Age: *F*(1,123) = 3.22, *p* = .075  Night waking number: *F*(1,132) = 0.37, *p* = .545  Age x Night waking number: *F*(1,130) = 0.59, *p* = .446 | | | |
| **Sleep measure: Diary** |  |  |  |  |
| **ASQ: Problem-solving subscale** |  |  |  |  |
| Night sleep duration |  |  |  |  |
| M1 | 1048.41 | 1060.41 | 1078.11 | 6 |
|  | Age: *F*(1,110) = 11.83, *p* = .001  Day sleep duration: *F*(1,111) = 0.43, *p* = .515 | | | |
| M2 | 1046.94 | 1060.94 | 1081.58 | 7 |
|  | Age: *F*(1,119) = 0.84, *p* = .361  Day sleep duration: *F*(1,124) = 0.82, *p* = .368  Age x day sleep duration: *F*(1,120) = 1.50, *p* = .223 | | | |
| Day sleep duration |  |  |  |  |
| M1 | 1040.81 | 1052.81 | 1070.46 | 6 |
|  | Age: *F*(1,114) = 12.81, *p* = .001  Day sleep duration: *F*(1,130) = 0.44, *p* = .51 | | | |
| M2 | 1033.35 | 1047.35 | 1067.94 | 7 |
|  | Age: *F*(1,128) = 1.55, *p* = .216  Day sleep duration: *F*(1,136) = 4.97, *p* = .027  Age x day sleep duration: *F*(1,131) = 7.77, *p* = .006 | | | |
| WASO |  |  |  |  |
| M1 | 1012.98 | 1024.98 | 1042.50 |  |
|  | Age: *F*(1,107) = 14.97, *p* = .0001  WASO: *F*(1,127) = 0.19, *p* = .668 | | | |
| M2 | 1012.06 | 1026.06 | 1046.50 |  |
|  | Age: *F*(1,119) = 10.11, *p* = .002  WASO: *F*(1,129) = 1.11, *p* = .294  Age x WASO: *F*(1,131) = 0.93, *p* = .34 | | | |
| Night waking number |  |  |  |  |
| M1 | 1046.01 | 1058.01 | 1075.70 |  |
|  | Age: *F*(1,112) = 13.62, *p* < .001  Night waking number: *F*(1,116) = 0.08, *p* = .778 | | | |
| M2 | 1043.69 | 1057.69 | 1078.34 |  |
|  | Age: *F*(1,118) = 11.50, *p* = .001  Night waking number: *F*(1,127) = 2.07, *p* = .153  Age x Night waking number: *F*(1,124) = 2.39, *p* = .124 | | | |
| **Sleep measure: BISQ** |  |  |  |  |
| **ASQ Problem-solving subscale** |  |  |  |  |
| Night sleep duration |  |  |  |  |
| M1 | 1201.39 | 1213.39 | 1231.85 |  |
|  | Age: *F*(1,121) = 13.77, *p* < .001  Night sleep duration: *F*(1,159) = 0.07, *p* = .79 | | | |
| M2 | 1200.56 | 1214.56 | 1236.09 |  |
|  | Age: *F*(1,156) = 1.95, *p* = .17  Night sleep duration: *F*(1,155) = 0.92, *p* = .34  Age x Night sleep duration: *F*(1,156) = 0.85, *p* = .36 | | | |
| Day sleep duration |  |  |  |  |
| M1 | 1184.57 | 1196.57 | 1214.99 | 6 |
|  | Age: *F*(1,124) = 16.54, *p* < .001  Day sleep duration: *F*(1,154) = 7.02, *p* = .89 | | | |
| M2 | 1175.63 | 1189.63 | 1211.11 | 7 |
|  | Age: *F*(1,137) = 0.50, *p* = .481  Day sleep duration: *F*(1,150) = 7.69, *p* = .006  Age x day sleep duration: *F*(1,148) = 9.28, *p* = .003 | | | |
| WASO |  |  |  |  |
| M1 | 1184.05 | 1196.05 | 1214.42 |  |
|  | Age: *F*(1,122) = 11.36, *p* = .001  Day sleep duration: *F*(1,152) = 2.59, *p* = .110 | | | |
| M2 | 1183.98 | 1197.98 | 1219.42 |  |
|  | Age: *F*(1,126) = 5.01, *p* = .027  WASO: *F*(1,142) = 0.64, *p* = .426  Age x WASO: *F*(1,136) = 0.07, *p* = .794 | | | |
| Night waking number |  |  |  |  |
| M1 | 1200.76 | 1212.76 | 1231.21 |  |
|  | Age: *F*(1,118) = 13.68, *p* < .001  Night waking number: *F*(1,132) = 0.72, *p* = .399 | | | |
| M2 | 1200.37 | 1214.37 | 1235.90 |  |
|  | Age: *F*(1,121) = 6.78, *p* = .010  Night waking number: *F*(1,143) = 0.14, *p* = .711  Age x Night waking number: *F*(1,142) = 0.40, *p* = .527 | | | |

| *Table SM6e.* Linear mixed model statistics for the association of ASQ and sleep parameters of three methods (Actigraphy, diary, BISQ) – Social subscale | | | | |
| --- | --- | --- | --- | --- |
|  | **-2LL** | **AIC** | **BIC** | **df** |
| **Sleep measure: Actigraphy** | | | | |
| **ASQ: Social subscale** | | | | |
| Night sleep duration |  |  |  |  |
| M1 | 1102.44 | 1114.44 | 1132.26 | 6 |
|  | Age: *F*(1,101) = 1.09, *p* = .299  Day sleep duration: *F*(1,127) = 1.00, *p* = .319 | | | |
| M2 | 1101.25 | 1115.25 | 1136.04 | 7 |
|  | Age: *F*(1,139) = 0.83, *p* = .364  Day sleep duration: *F*(1,130) = 1.91, *p* = .170  Age x day sleep duration: *F*(1,139) = 1.21, *p* = .274 | | | |
| Day sleep duration |  |  |  |  |
| M1 | 1065.54 | 1077.54 | 1095.14 | 6 |
|  | Age: *F*(1,102) = 1.50, *p* = .223  Day sleep duration: *F*(1,1327) = 2.67, *p* = .104 | | | |
| M2 | 1063.86 | 1077.86 | 1098.40 | 7 |
|  | Age: *F*(1,130) = 2.57, *p* = .111  Day sleep duration: *F*(1,134) = 2.90, *p* = .09  Age x day sleep duration: *F*(1,133) = 1.69, *p* = .196 | | | |
| WASO |  |  |  |  |
| M1 | 1129.58 | 1141.58 | 1159.56 | 6 |
|  | Age: *F*(1,103) = 1.85, *p* = .177  WASO: *F*(1,139) = 2.43, *p* = .121 | | | |
| M2 | 1129.50 | 1143.50 | 1164.48 | 7 |
|  | Age: *F*(1,138) = 0.02, *p* = .878  WASO: *F*(1,137) = 0.80, *p* = .372  Age x WASO: *F*(1,131) = 0.08, *p* = .774 | | | |
| Night waking number |  |  |  |  |
| M1 | 1109.12 | 1121.12 | 1138.98 | 6 |
|  | Age: *F*(1,100) = 0.50, *p* = .482  Night waking number: *F*(1,128) = 1.04, *p* = .311 | | | |
| M2 | 1109.10 | 1123.10 | 1143.93 | 7 |
|  | Age: *F*(1,122) = 0.13, *p* = .715  Night waking number: *F*(1,131) = 0.05, *p* = .825  Age x Night waking number: *F*(1,130) = 0.02, *p* = .885 | | | |
| **Sleep measure: Diary** |  |  |  |  |
| **ASQ: Social subscale** |  |  |  |  |
| **Night sleep duration** |  |  |  |  |
| M1 | 1054.37 | 1066.37 | 1084.02 | 6 |
|  | Age: *F*(1,99) = 1.26, *p* = .265  Day sleep duration: *F*(1,123) = 0.04, *p* = .844 | | | |
| M2 | 1054.07 | 1068.07 | 1088.66 | 7 |
|  | Age: *F*(1,121) = 0.20, *p* = .655  Day sleep duration: *F*(1,121) = 0.34, *p* = .559  Age x day sleep duration: *F*(1,120) = 0.305, *p* = .582 | | | |
| Day sleep duration |  |  |  |  |
| M1 | 1046.60 | 1058.60 | 1076.21 | 6 |
|  | Age: *F*(1,106) = 2.50, *p* = .117  Day sleep duration: *F*(1,135) = 0.95, *p* = .331 | | | |
| M2 | 1042.33 | 1056.33 | 1076.87 | 7 |
|  | Age: *F*(1,123) = 1.67, *p* = .199  Day sleep duration: *F*(1,132) = 2.12, *p* = .148  Age x day sleep duration: *F*(1,125) = 4.36, *p* = .039 | | | |
| WASO |  |  |  |  |
| M1 | 1019.93 | 1031.93 | 1049.41 | 6 |
|  | Age: *F*(1,94) = 3.04, *p* = .085  Day sleep duration: *F*(1,131) = 3.93, *p* = .05 | | | |
| M2 | 1019.89 | 1033.89 | 1054.28 | 7 |
|  | Age: *F*(1,114) = 0.94, *p* = .334  Day sleep duration: *F*(1,125) = 0.29, *p* = .592  Age x day sleep duration: *F*(1,130) = 0.04, *p* = .848 | | | |
| Night waking number |  |  |  |  |
| M1 | 1055.43 | 1067.43 | 1085.08 | 6 |
|  | Age: *F*(1,99) = 1.55, *p* = .215  Night waking number: *F*(1,120) = 0.04, *p* = .837 | | | |
| M2 | 1055.40 | 1069.40 | 1090.00 | 7 |
|  | Age: *F*(1,111) = 0.35, *p* = .558  Night waking number: *F*(1,122) = 0.04, *p* = .842  Age x Night waking number: *F*(1,121) = 0.02, *p* = .876 | | | |
| **Sleep measure: BISQ** |  |  |  |  |
| **ASQ: Social subscale** |  |  |  |  |
| Night sleep duration |  |  |  |  |
| M1 | 1211.30 | 1223.30 | 1241.71 | 6 |
|  | Age: *F*(1,110) = 1.58, *p* = .21  Night sleep duration: *F*(1,159) = 0.23, *p* = .63 | | |  |
| M2 | 1211.04 | 1225.04 | 1246.52 |  |
|  | Age: *F*(1,153) = 0.47, *p* = .50  Night sleep duration: *F*(1,151) = 0.42, *p* = .518  Age x Night sleep duration: *F*(1,153) = 0.27, *p* = .606 | | | |
| Day sleep duration |  |  |  |  |
| M1 | 1202.96 | 1214.96 | 1233.33 | 6 |
|  | Age: *F*(1,117) = 0.53, *p* = .466  Day sleep duration: *F*(1,156) = 1.94, *p* = .17 | | | |
| M2 | 1202.36 | 1216.36 | 1237.80 | 7 |
|  | Age: *F*(1,137) = 0.10, *p* = .749  Day sleep duration: *F*(1,149) = 1.54, *p* = .217  Age x day sleep duration: *F*(1,150) = 0.60, *p* = .440 | | | |
| WASO |  |  |  |  |
| M1 | 1196.46 | 1208.46 | 1226.79 | 6 |
|  | Age: *F*(1,111) = 1.23, *p* = .270  WASO: *F*(1,155) = 1.57, *p* = .212 | | | |
| M2 | 1196.44 | 1210.44 | 1231.83 | 7 |
|  | Age: *F*(1,123) = 0.81, *p* = .370  WASO: *F*(1,146) = 0.09, *p* = .765  Age x WASO: *F*(1,142) = 0.02, *p* = .889 | | | |
| Night waking number |  |  |  |  |
| M1 | 1211.51 | 1223.51 | 1241.93 |  |
|  | Age: *F*(1,108) = 1.86, *p* = .176  Night waking number: *F*(1,139) = 0.02, *p* = .894 | | | |
| M2 | 1211.45 | 1225.45 | 1246.94 |  |
|  | Age: *F*(1,119) = 0.33, *p* = .569  Night waking number: *F*(1,143) = 0.04, *p* = .844  Age x Night waking number: *F*(1,1142) = 0.06, *p* = .808 | | | |
